# A point mutation in the kinase domain of CRK10 leads to xylem vessel collapse and activation of defence responses in Arabidopsis

**DOI:** 10.1093/jxb/erad080

**Published:** 2023-03-03

**Authors:** Maiara Piovesana, Ana K M Wood, Daniel P Smith, Michael J Deery, Richard Bayliss, Esther Carrera, Nikolaus Wellner, Ondrej Kosik, Johnathan A Napier, Smita Kurup, Michaela C Matthes

**Affiliations:** Department of Plant Sciences, Rothamsted Research, Harpenden AL5 2JQ, UK; College of Life and Environmental Sciences, Streatham Campus, Exeter EX4 4PY, UK; Department of Biointeractions and Crop Protection, Rothamsted Research, Harpenden AL5 2JQ, UK; Department of Computational and Analytical Sciences, Rothamsted Research, Harpenden AL5 2JQ, UK; Cambridge Centre for Proteomics, University of Cambridge, Cambridge CB2 1QR, UK; School of Molecular and Cellular Biology, Faculty of Biological Sciences, University of Leeds, Leeds LS2 9JT, UK; Instituto de Biología Molecular y Celular de Plantas, Universidad Politècnica de València, Valencia 46022, Spain; Quadram Institute, Norwich Research Park, Norwich NR4 7UQ, UK; Department of Plant Sciences, Rothamsted Research, Harpenden AL5 2JQ, UK; Department of Plant Sciences, Rothamsted Research, Harpenden AL5 2JQ, UK; Department of Plant Sciences, Rothamsted Research, Harpenden AL5 2JQ, UK; Department of Plant Sciences, Rothamsted Research, Harpenden AL5 2JQ, UK; Queen’s University, Canada

**Keywords:** αC helix, Arabidopsis, cysteine-rich receptor-like kinase CRK10, defence response, *Fusarium oxysporum*, gain-of-function mutation, kinase regulation, transcriptomics, xylem vessel collapse

## Abstract

Cysteine-rich receptor-like kinases (CRKs) are a large family of plasma membrane-bound receptors ubiquitous in higher plants. However, despite their prominence, their biological roles have remained largely elusive so far. In this study we report the characterization of an Arabidopsis mutant named *crk10-A397T* in which alanine 397 has been replaced by a threonine in the αC helix of the kinase domain of CRK10, known to be a crucial regulatory module in mammalian kinases. The *crk10-A397T* mutant is a dwarf that displays collapsed xylem vessels in the root and hypocotyl, whereas the vasculature of the inflorescence develops normally. *In situ* phosphorylation assays with His-tagged wild type and *crk10-A397T* versions of the CRK10 kinase domain revealed that both alleles are active kinases capable of autophosphorylation, with the newly introduced threonine acting as an additional phosphorylation site in *crk10-A397T*. Transcriptomic analysis of wild type and *crk10-A397T* mutant hypocotyls revealed that biotic and abiotic stress-responsive genes are constitutively up-regulated in the mutant, and a root-infection assay with the vascular pathogen *Fusarium oxysporum* demonstrated that the mutant has enhanced resistance to this pathogen compared with wild type plants. Taken together our results suggest that *crk10-A397T* is a gain-of-function allele of *CRK10*, the first such mutant to have been identified for a CRK in Arabidopsis.

## Teaser

A gain-of-function allele of the cysteine-rich receptor-like kinase CRK10 (*crk10-A397T*) causes defects in the vasculature and activates defence pathways in hypocotyls and roots of Arabidopsis.

## Introduction

Plant growth and development are modulated by a multitude of intrinsic growth regulators and environmental cues. Factors regulating development as well as environmental and pathogenic signals are mostly recognized by receptor-like kinases (RLKs), membrane-localized receptors that perceive and transduce these signals to the intracellular environment. Similar to animal receptor tyrosine kinases (RTKs), these receptors consist of an extracellular domain that perceives specific ligands, a single-pass transmembrane domain, and a cytoplasmic kinase domain that transduces the signal via phosphorylation of downstream target proteins in the cytoplasm in order to tailor a cellular response (Shiu and Bleecker, 2001; De Smet *et al.*, 2009). Their highly variable extracellular domains are used for the classification of RLKs into subgroups, the largest of which (~200 genes in Arabidopsis) is characterized by leucine-rich repeats (LRR-RLKs) (Diévart and Clark, 2004). The well-studied brassinosteroid receptor BRASSINOSTEROID INSENSITIVE 1 (BRI1; AT4G39400) and the microbial pattern recognition receptors (PRR) FLAGELLIN SENSING 2 (FLS2; AT5G46330) and EF-TU RECEPTOR (EFR; AT5G20480), for example, are well-characterized members of this subgroup (Friedrichsen *et al.*, 2000; Chinchilla *et al.*, 2006; Zipfel *et al.*, 2006).

Among the multiple subfamilies of RLKs found in plant genomes, the one containing the cysteine-rich receptor-like kinases (CRKs) is one of the largest with over 40 members in Arabidopsis. The signature motif for CRKs is the presence of, in most cases, two repeats of the DOMAIN OF UNKNOWN FUNCTION 26 (DUF26) in their extracellular domain, which contains three cysteine residues in the conserved configuration C-X8-C-X2-C (Chen, 2001). Although the functional significance of the DUF26 domain remains to be elucidated, it was originally suggested to participate in redox sensing (Wrzaczek *et al.*, 2010; Bourdais *et al.*, 2015). More recent data obtained from the crystallographic analysis of the DUF26-containing ectodomain of the plasmodesmata-localized proteins PDLP5 and PDLP8, however, point towards the involvement of the cysteine residues in forming disulfide bonds for the structural stabilization of the protein rather than redox regulation (Vaattovaara *et al.*, 2019). The same study also revealed that the DUF26 domain shows strong structural similarity to fungal carbohydrate-binding lectins, which suggests that DUF26-containing proteins might constitute a group of carbohydrate-binding proteins in plants (Vaattovaara *et al.*, 2019). Corroborating this hypothesis, the ability to interact with carbohydrates was demonstrated for the secreted DUF26-containing antifungal proteins Ginkbilobin2 (Gnk2) from *Gingko biloba* (Miyakawa et al., 2009, 2014) and Anti-Fungal Protein 1 (AFP1) from maize (Ma *et al.*, 2018), both of which bind to the monosaccharide mannose, a component of fungal cell walls. Bona fide ligands for CRKs, however, still remain to be identified.

Despite the large number of CRKs among the RLK superfamily, very little is known about their specific biological roles and the regulation of downstream signalling events. Efforts to assign functions to members of this family have involved a comprehensive analysis of a collection of T-DNA knockout lines for 41 CRKs of Arabidopsis, which suggested a role for several members in the fine-tuning of stress adaptation and plant development (Bourdais *et al.*, 2015). Most knockout lines, however, did not display obvious phenotypes, as is expected for a large gene family due to redundancy amongst its members. Studies in Arabidopsis also revealed that several CRKs are transcriptionally regulated by a wide variety of biotic and abiotic factors such as ozone, ultraviolet light, reactive oxygen species, the hormone salicylic acid (SA), and elicitation with pathogen-derived molecules (Czernic *et al.*, 1999; Du and Chen, 2000; Ohtake *et al.*, 2000; Wrzaczek *et al.*, 2010).

Functionally, CRKs belong to the RD subclass of Ser/Thr kinases (Vaattovaara *et al.*, 2019), which typically carry a conserved arginine (R) immediately preceding the invariant aspartate (D) in subdomain VI required for catalytic activity, and are, in most cases, activated through autophosphorylation of the activation loop (Nolen *et al.*, 2004). Although the ability to autophosphorylate as well as to phosphorylate substrates *in vitro* has been demonstrated for CRK2 and CRK7 (Idänheimo *et al.*, 2014; Kimura *et al.*, 2020), for example, detailed studies investigating the role of specific phosphorylation sites for the regulation of kinase activity are still outstanding for CRKs.

Establishing ligand–receptor pairs is not a trivial task and exploring regulation and function of RLKs without the knowledge of their activating ligands poses a challenge. In these circumstances, mutants harbouring gain-of-function alleles of RLKs, where kinase activity occurs in the absence of the ligand, can be a useful resource to study receptor regulation and function. In this study we describe the characterization of one such gain-of-function allele, *crk10-A397T*, obtained for *CYSTEINE-RICH RECEPTOR-LIKE KINASE 10* (*CRK10*; AT4G23180) of Arabidopsis. This mutation leads to the replacement of alanine 397 with threonine in the αC helix of the kinase domain of the protein, with the newly introduced Thr397 acting as an additional phosphorylation site *in situ*. We show that the *crk10-A397T* allele causes a dwarf phenotype in Arabidopsis, which is associated with the collapse of xylem vessels in roots and hypocotyls. Analysis of the transcriptome shows the occurrence of extensive transcriptional re-programming of immune- and cell wall-related genes in the hypocotyls of the mutant plants. Biochemical analyses reveal that these transcriptional changes are accompanied by alterations in cell wall composition. A pathogen assay indicates enhanced resistance to the soil-borne vascular pathogen *Fusarium oxysporum*. Taken together our results suggest that *crk10-A397T* is a gain-of-function allele of CRK10 that leads to xylem vessel collapse and the activation of defence responses to pathogens.

## Materials and methods

### Plant materials, growth conditions, and micrografting

Arabidopsis ecotype Col-0 plants were grown in Grobanks cabinets (CLF 2006, Plant Climatics, Germany) in Levington F2 + Sand compost in long day conditions (16 h/8 h), 23/18 ºC day/night temperature, and 200 µmol m^−2^ s^−1^ light intensity. For *in vitro* experiments, surface-sterilized seeds were cultivated on ½ Murashige and Skoog (MS) plates (Duchefa Biochemie). The T-DNA lines SAIL_427_E09 and SALK_116653 were obtained from the Nottingham Arabidopsis Stock Centre. Micrografting was performed according to the procedure described by Turnbull *et al.* (2002). Successful grafts were transferred to soil 7–10 d post-grafting.

### RNA isolation, RNA-Seq library construction, and sequencing

Total RNA was extracted from hypocotyls of Arabidopsis plants using the RNeasy Mini Kit (Qiagen). Four biological replicates were isolated per genotype per time point with each biological replicate consisting of a pool of 50–60 hypocotyls. Samples were treated with DNase Turbo DNA-free kit (Thermo Fisher Scientific). Prior to library preparation RNA quality was assessed on a 2100 Bioanalyzer (Agilent Technologies). Library preparation and paired-end sequencing was performed by Exeter Sequencing Service (University of Exeter, UK) using the Illumina HiSeq 125 PE sequencing platform.

### RNA-Seq analysis

Using the Galaxy (https://usegalaxy.org/) bioinformatics web pipeline, the quality of the reads was assessed using MultiQC (https://multiqc.info/), reads were trimmed using Trimmomatic (Bolger *et al.*, 2014), and mapped with HiSAT2 (Kim *et al.*, 2015; https://daehwankimlab.github.io/hisat2/). The table of counts was acquired using the featureCounts functions in the Subread (Liao *et al.*, 2019; https://bioconductor.org/packages/release/bioc/html/Rsubread.html), on the R Bioconductor platform (https://bioconductor.org/). Genes that did not meet a threshold of 5 counts in at least three out of four biological replicates were discarded. Differential expression analysis was performed (using the default Wald test) in the R (v3.6.1) Bioconductor package DESeq2 (Love *et al.*, 2014; https://bioconductor.org/packages/release/bioc/html/DESeq2.html). Gene Ontology (GO) enrichment analysis (Single Enrichment Tool, AgriGO v2.0; Tian *et al.*, 2017) and similarity comparison with deposited micro-array datasets (Signature tool, GENEVESTIGATOR, Hruz *et al.*, 2008) were performed for the set of 274 core differentially expressed genes (DEGs).

### Quantification of transcript abundance by quantitative PCR

RNA was isolated using TRI Reagent (Merck) and treated with DNase I, Amplification Grade (Thermo Fisher Scientific) prior to cDNA synthesis with SuperScript III Reverse Transcriptase (Thermo Fisher Scientific). All qPCR reactions were performed in a LightCycler 96 Real-Time PCR System (Roche Diagnostics) using the FastStart Essential DNA Green Master (Roche Diagnostics). Primers AtCRK10 forward (For)/reverse (Rev) were used for quantification of *CRK10* expression, and primers AtACT2 For/Rev (*ACTIN2*; AT3G18780) and AtUBC21 For/Rev (*UBC21*; AT5G25760) were used as internal controls (see Supplementary Table S1). The $2_{}^{-\Delta \Delta C_{t}}$ method (Livak and Schmittgen, 2001) was used to calculate relative expression.

### Quantification of plant hormones

Three replicates, each containing between 75 and 100 mg (fresh weight) of hypocotyls isolated from 3-week-old wild type (WT) and mutant plants were prepared. Hormone analysis was performed using the platform provided by The Plant Hormone Quantification Service from Universitat Politècnica de València according to their protocol (https://ibmcp.upv.es/services/plant-hormone-quantification/).

### Generation of genetic constructs

The cDNA clone (U60398) containing full length *CRK10* was obtained from the Arabidopsis Biological Resource Center (https://abrc.osu.edu/). Vector pJD330 was kindly provided by Dr D. R. Gallie, and vector RS 3GSeedDSRed MCS by Edgar B. Cahoon, University of Nebraska-Lincoln. A list of primers used in this study can be found in Supplementary Table S1. The 35S:*CRK10*-NOSt construct was generated by amplification of the full-length cDNA of *CRK10* from clone U60398 with primers CRK10 *Sal*I For and CRK10 *Sac*I Rev. The CRK10 cDNA sequence was subcloned into pJD330 between the 35S promoter and NOS terminator after removal of the β-glucuronidase (GUS)-containing fragment by restriction digestion (pJD330 35S:*CRK10*-NOSt). The 35S:*CRK10*-NOSt fragment was then amplified with 35S *Asc*I For and NOSt *Asc*I Rev primers and inserted into the binary vector RS 3GSeedDSRed MCS. The *CRK10*_Pro_:*crk10-A397T*-NOSt construct was generated by replacing the 35S promoter in pJD330 35S:*CRK10*-NOSt with 1 kb of the native promoter of *CRK10* (*CRK10*_Pro_) obtained by amplification of genomic DNA with primers CRK10 Pro *Sph*I For and CRK10 Pro *Sal*I Rev. *In vitro* mutagenesis (GeneArt; Thermo Fisher Scientific) with primers CRK10 A397T For and CRK10 A397T Rev was used to introduce the G>A mutation responsible for the replacement of A397 by T. CRK10 Pro *Asc*I For and NOSt *Asc*I Rev primers were used to amplify *CRK10*_Pro_:*crk10-A397T*-NOSt for cloning into the RS 3GSeedDSRed MCS binary vector. The *CRK10*_Pro_:*GUS*-NOSt construct was generated by replacing 35S in pJD330 with the *CRK10*_Pro_ sequence obtained by amplification with primers CRK10 Pro *Sph*I For and CRK10 Pro *Nco*I Rev through restriction digestion. The *CRK10*_Pro_:*GUS*:NOSt fusion was amplified with primers CRK10 Pro *Asc*I For and NOSt *Asc*I Rev for transfer into RS 3GSeedDSRed MCS. The translational fusion of *CRK10* with *mCherry* was obtained by replacing the stop codon of *CRK10* in pJD330 35S:*CRK10*-NOSt with a *Sac*I restriction site (primers CRK10 wsc *Sac*I For and CRK10 wsc *Sac*I Rev) by *in vitro* mutagenesis. This restriction site was used to insert in frame the *mCherry* sequence that had been amplified with compatible primers (mCherr*y Sac*I For and mCherry *Sac*I Rev). The *CRK10-mCherry*-NOSt fragment was then amplified with primers CRK10 *Sal*I For and NOSt *Not*I Rev and cloned into the pENTR1A Dual Selection Vector (Thermo Fisher Scientific). Gateway cloning (Thermo Fisher Scientific) into destination vector pB2GW7 (Karimi *et al.*, 2002) generated the final construct 35S:*CRK10-mCherry*-NOSt.

### Transformation of plants

Detection of transient expression of fluorescent fusions was performed by infiltration of *Nicotiana benthamiana* leaves according to Sparkes *et al.* (2006). Expression of fusion proteins was observed 72 h post-infiltration. In order to generate stable transformed lines Arabidopsis Col-0 was transformed by floral dip (Clough and Bent, 1998) with *Agrobacterium tumefaciens* GV3101 containing the respective constructs.

### Microscopy

#### Light microscopy

Thin sections were prepared by fixing plant tissue in 4% paraformaldehyde–2.5% glutaraldehyde followed by gradual dehydration with ethanol and infiltration with LRWhite resin (Agar Scientific). Sections were prepared using a Reichert ultramicrotome (section thickness: 1–2 µm) and stained with 0.5% potassium permanganate. Thin sections were observed with a Zeiss Axiophot microscope equipped with a Q-Imaging Retiga EXi CCD camera (QImaging, Canada).

#### Confocal microscopy

Confocal microscopy was performed using the Zeiss 780 LSM system. For detection of the autofluorescence of lignin, non-stained resin-embedded thin sections were imaged with an excitation wavelength of 405 nm and emission was collected at 451–480 nm and 560–612 nm. mCherry fluorescence from transiently transformed *N. benthamiana* leaves or stably transformed Arabidopsis hypocotyls was detected with a laser excitation wavelength of 561 nm and collection of emission at 578–639 nm. Plasmolysis was performed using a 0.8 M mannitol solution for 40 min.

#### Transmission electron microscopy

Samples were prepared by fixing plant material by high-pressure freezing using a Leica HPM100, followed by freeze substitution with 100% ethanol (Leica EM Auto Freeze substitution) and infiltration with LRWhite resin (Agar Scientific). Ultra-thin sections were prepared with a Leica EM UCT ultramicrotome (section thickness: 90 nm) and were collected on pioloform/carbon-coated nickel grids (Agar Scientific) and stained with 2.5% uranyl acetate and Reynolds lead citrate (Reynolds, 1963). Ultrathin sections were imaged using a JEOL-2100Plus transmission electron microscope (JEOL, Japan) equipped with a Gatan OneView IS camera (Gatan, USA).

### Fourier-transform infrared spectroscopy

Transverse cross sections of hypocotyls of 3-week-old WT and *crk10-A397T* mutant plants for Fourier-transform infrared spectroscopy (FTIR) analysis were prepared using a cryostat (CM1850 Cryostat, Leica Microsystems; section thickness: 20 µm) with three replicates per sample. Cross sections were washed with 70% ethanol and air-dried on barium fluoride (BaF_2_) discs prior to analysis using a Nicolet iN10MX infrared microscope (Thermo Scientific) equipped with a ×15 infrared (IR) objective. FTIR maps were obtained by transmission aperture mapping with a mercury–cadmium–telluride detector and an *x–y* step size of 10 μm (20 × 20 μm^2^ aperture). A total of 128 scans were averaged at 8 cm^−1^ for each image pixel. An empty spot on the BaF_2_ disk was used as background. The maps were exported in ENVI format and processed in MATLAB (The MathWorks, Inc.). The spectra were truncated to 1800–700 cm^−1^ and the density map was calculated by averaging the IR absorption from 1800 to 800 cm^−1^. Characteristic band intensities for the following components were mapped to visualize their distribution: 1018 cm^−1^ for pectin, 1033 cm^−1^ and 1050 cm^−1^ for hemicelluloses and cellulose, 1511 cm^−1^ for lignin, 1650 cm^−1^ for protein, and 1735 cm^−1^ for ester groups.

### Quantification of total monosaccharides

Thirty hypocotyls of 3-week-old WT and *crk10-A397T* mutant plants were collected per replicate with three replicates per sample. Alcohol insoluble residue (AIR) preparation was performed according to Goubet *et al.* (2009). A total of 200–600 µg of AIR per sample was hydrolysed in 2 M trifluoroacetic acid (Sigma-Aldrich) prior to quantification of acidic and neutral monosaccharides by high performance anion-exchange chromatography with pulsed amperometric detection. Acidic monosaccharides (glucuronic and galacturonic acid) were quantified using a Dionex ICS-3000 ion chromatography system (Thermo Scientific) and CarboPac PA-200 columns (Thermo Scientific). For the quantification of neutral monosaccharides, a Dionex ICS-5000+ equipped with eluent generator (Thermo Scientific) and CarboPac PA-20 columns (Thermo Scientific) were used. Chromeleon analytical software (version 7.2SR5; Thermo Scientific) was used for peak marking and quantification.

### Histochemical analysis of β-glucuronidase activity

Plant tissue was incubated overnight in X-gluc (Melford) solution at 37 °C. Chlorophyll was removed with 80% ethanol prior to imaging using a Leica M205 FA stereomicroscope (Leica Microsystems).

### Recombinant protein expression, purification, and analysis

Primers used to generate the 6× His-tagged CRK10 kinase domain (KD) constructs are listed in Supplementary Table S1. The KD of *CRK10* was amplified with primers CRK10 KD *Sal*I For and CRK10 KD *Not*I Rev and cloned into pENTR1A Dual Selection Vector (Thermo Fisher Scientific). *In vitro* mutagenesis was used to generate the gain-of-function mutation of CRK10 (primers CRK10 A397T For and CRK10 A397T Rev) and the dead kinase variant (primers CRK10 D473N For and CRK10 D473N Rev) prior to Gateway cloning with pDEST17 (Thermo Fisher Scientific). The 6× His tagged proteins were expressed in BL21 AI One-Shot *Escherichia coli* cells (Thermo Fisher Scientific). Bacterial cultures were grown to an OD_600_ of 0.4–0.5 after which protein expression was induced by adding l-arabinose to a final concentration of 0.2%. After 3 h bacterial cells were lysed by sonication, His-tagged proteins were purified with the HIS-Select Nickel Affinity Gel (Sigma-Aldrich) and protein concentration determined by the Bradford method (Protein Assay Dye Reagent, Bio-Rad). For phosphatase treatment, 5 µg protein extract was treated with Lambda Protein Phosphatase (Lambda PP, New England BioLabs) for 1 h 30 min at 30 ºC. Samples were resolved by SDS-PAGE (NuPAGE 4-12% Bis-Tris Protein Gels, Thermo Fisher Scientific) and gels were stained with Quick Coomassie Stain (Generon). For western blotting, proteins were transferred to a polyvinylidene difluoride membrane (iBlot Transfer Stack, PVDF, Thermo Fisher Scientific) and hybridized with a His-probe (H-3) horseradish peroxidase monoclonal antibody (Santa Cruz Biotechnology). The membrane was washed and incubated with Amersham ECL Western Blotting Detection Reagent (GE Healthcare) according to manufacturer’s instructions.

### Determination of phosphorylation sites

Protein bands corresponding to His-CRK10kd^WT^ and His-CRK10kd^A397T^ were excised from an acrylamide gel and sent to the Cambridge Centre for Proteomics, (https://proteomics.bio.cam.ac.uk/core-facility) for analysis by LC-MS/MS. Data were submitted to the Mascot search algorithm (Matrix Science, London UK, version 2.6.0) against a custom database consisting of the CRK10^WT^ and CRK10^A397T^ sequences and the UniProt Arabidopsis database (41552 sequences; 17578843 residues). A significance threshold value of *P*<0.05 and a peptide cut-off score of 20 were applied.

### Protein modelling

A structural model of the kinase domain of CRK10 was generated by homology modelling using PyMOD 3.0 with default parameters (Janson *et al.*, 2017). The kinase domain of BRI1 (PDB code 5LPV) (Bojar *et al.*, 2014) was used as template retaining the ATP analogue (phosphoaminophosphonic acid adenylate ester) but no other heteroatoms.

### *Fusarium oxysporum* infection assay

Susceptibility to infection by *Fusarium oxysporum* f. sp*. conglutinans* 699 (kindly provided by Prof. Antonio Di Pietro) was assessed by a root infection assay according to Masachis *et al.* (2016) with minor modifications. Arabidopsis seedlings (12 d old) grown *in vitro* were inoculated by immersing their roots for 20 min in a suspension of 1 × 10^6^ microconidia ml^−1^ of *F. oxysporum* f. sp*. conglutinans* 699. Subsequently seedlings were transferred to soil and cultivated in a growth chamber under long day conditions and a temperature set at 28 ºC during the day and 25 ºC at night. For each repetition of the experiment, 80 plants per genotype were arranged in a randomized blocked tray design, and mortality was assessed daily between 7 and 20 d post-inoculation. The experiment was performed twice. To determine fungal burden, seedlings were inoculated as described above and sampled at 2 and 7 d post-inoculation. At these time points, total DNA was extracted (protocol adapted from Yu *et al.*, 2019) from a pool of eight seedlings per genotype and the relative amount of fungal DNA was quantified by qPCR using the primers *ACTIN1* For/Rev (*F. oxysporum*) and normalized to the Arabidopsis *ACTIN2* gene (primers AtACT2 For/Rev) (see Supplementary Table S1). The experiment was repeated three times. Results were expressed relative to WT at 2 d post-inoculation.

### Statistical analysis

Statistical tests were performed using Genstat software (Genstat for Windows 21st Edition; VSN International, Hemel Hempstead, UK). Student’s *t*-test was used to assess statistical differences between two variants. To assess whether the pattern of segregation of the dwarf phenotype followed the expected 1:2:1 ratio, the $\chi^{2}=\underset{i=1}{\overset{r}{\sum }}\;\frac{{\left(O_{i}-E_{i}\right)}^{2}}{E_{i}}$ chi-square statistic was used, where *O*_*i*_ is the observed count for group *i* and *E*_*i*_ is the expected count for group *i*. Under the null hypothesis of 1:2:1 segregation, this test statistic should follow a chi-square distribution with 2 degrees of freedom. The probability of survival of each genotype in the bioassay with *F. oxysporum* was assessed with a generalized linear model (Bernoulli distribution; logit link function fitted to the final mortality outcome of each plant); statistical significance of the genotypic effect was tested after removing variation associated with plant position within rows of different trays and quantified through a chi-square statistic of the difference in deviance. Statistical significance of the differences in fungal burden between genotypes was tested by ANOVA (expression levels were log-transformed to meet the ANOVA requirements, and each individual experiment was considered as a block).

## Results

### *crk10-A397T* is a semi-dominant mutant allele of *CRK10*

The Arabidopsis mutant characterized in this report was isolated in a forward genetic ethyl methanesulfonate screen performed for an unrelated study. In brief, six rounds of backcrosses to the WT Col-0 parent were performed in order to clean the genetic background of the mutant before the in-depth characterization. The homozygous mutant has a strong dwarf phenotype and observation of the segregating F_2_ progeny of the sixth backcross revealed the semi-dominant nature of the mutation, as WT, intermediate and dwarf phenotypes segregated according to a 1:2:1 ratio with heterozygous plants being clearly discernible (Supplementary Fig. S1A; χ_2_^2^=2.36, *P*=0.308). To determine the underlying mutation responsible for the dwarf phenotype, whole genome sequencing was performed on bulk segregants derived from the sixth backcross. This returned a list of 15 candidate genes containing point mutations in coding regions. We noticed that a point mutation (G>A) in the fourth exon of *CYSTEINE-RICH RECEPTOR-LIKE KINASE 10* (*CRK10*; AT4G23180) causes the replacement of alanine 397 by a threonine in the kinase domain of the protein (Supplementary Fig. S1B). As we considered this receptor-like kinase to be the most likely candidate among the 15 identified genes, we tested whether the dwarf phenotype could be rescued by constitutive expression of the WT cDNA sequence of *CRK10* under the control of the 35S promoter. All T_1_ transformants showed a WT phenotype (Supplementary Fig. S1C), suggesting that the correct gene had been identified. To further confirm that the mutation in *CRK10* causes the dwarf morphology, we recreated the G>A substitution by *in vitro* mutagenesis in the cDNA sequence of *CRK10* and introduced this open reading frame into a *crk10* knockout (KO) background (*crk10-2*, SAIL_427_E09, characterization of KO lines to follow) under the control of the 1 kb genomic region containing the putative native promoter of *CRK10.* A total of 25% of the recovered transformants were dwarfs, establishing a direct link between the dwarf phenotype and the mutant allele (Supplementary Fig. S1D). Subsequently, we will refer to this mutant as *crk10-A397T*.

### The *crk10-A397T* mutant is a dwarf

**Fig. 1.**
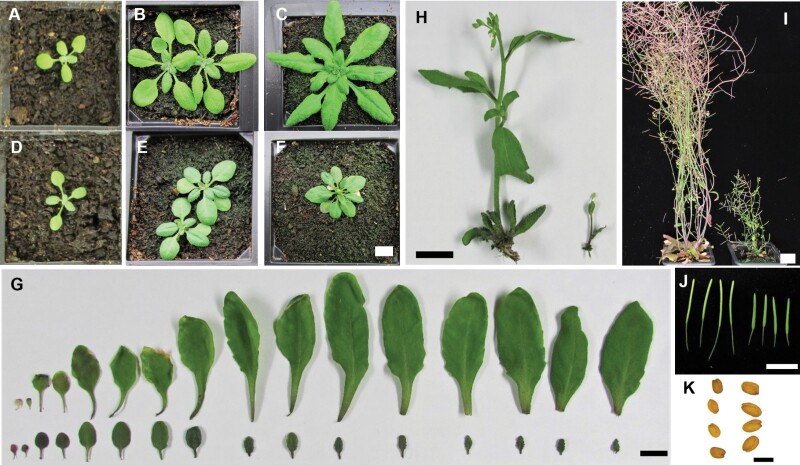
The *crk10-A397T* mutant is a dwarf. (A–F) Rosette morphology of WT (A–C) and *crk10-A397T* (D–F) plants at 2 (A, D), 3 (B, E), and 4 (C, F) weeks after sowing. Scale bar: 1 cm. (G) Leaf series of 4-week-old WT (top) and *crk10-A397T* (bottom) plants. Scale bar: 1 cm. (H) Main inflorescence stem of 5-week-old WT (left) and *crk10-A397T* (right) plants. Scale bar: 1 cm. (I) Ten-week-old WT (left) and *crk10-A397T* (right) plants. Scale bar: 2 cm. (J) Siliques of WT (left) and *crk10-A397T* (right) plants. Scale bar: 1 cm. (K) Seeds of WT (left) and *crk10-A397T* (right) plants. Scale bar: 500 µm.

WT Col-0 and *crk10*-*A397T* plants were phenotypically characterized for the duration of one entire life cycle. Although germination rate and establishment of seedlings were accelerated in the mutant (Supplementary Fig. S2A, B) these differences were no longer apparent 1 week after sowing. No other obvious differences in growth were observed between WT and *crk10*-*A397T* seedlings until week 2, after which leaf expansion became restricted in the *crk10-A397T* mutant and small, dark green leaves were formed causing a reduction of more than 70% in rosette size at 4 weeks after sowing (Fig. 1A–G; Supplementary Fig. S3A). Despite the dwarf phenotype during vegetative growth, the onset of flowering occurred simultaneously in *crk10-A397T* mutant and WT plants, although the main inflorescence remained stunted (Fig. 1H) as the shoot apical meristem was frequently aborted in the mutant (Supplementary Fig. S3B). At later stages, mutant plants developed numerous lateral inflorescences with smaller, stunted siliques filled with viable seeds that are in general larger than those of WT plants (Fig. 1I–K).

### The *crk10*-*A397T* mutant has collapsed xylem vessels in roots and hypocotyls

**Fig. 2.**
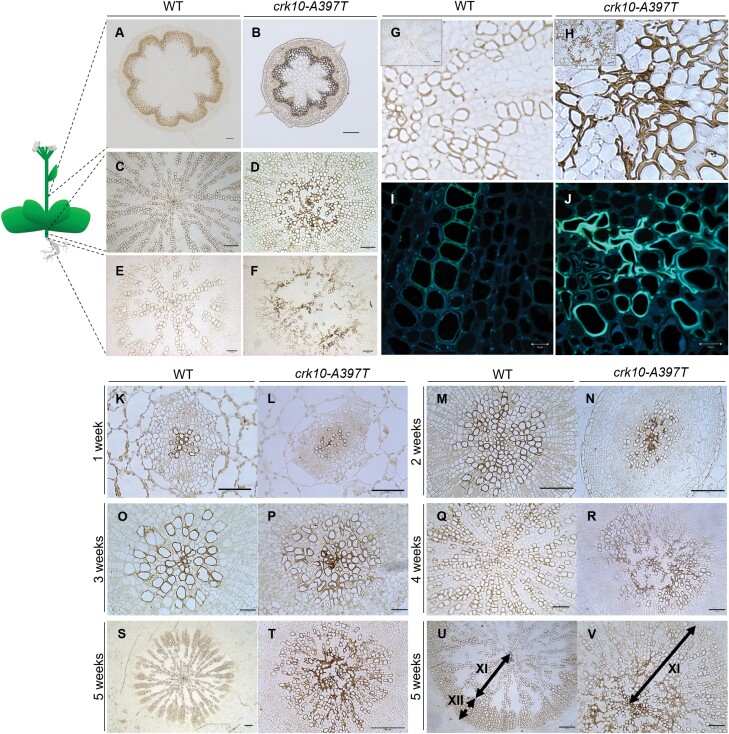
Xylem vessels collapse in the root and hypocotyl of *crk10-A397T* plants, but not in the stem. (A–F) Transverse cross sections of the base of stem (A, B), hypocotyl (C, D), and roots (E, F) of 5-week-old WT (A, C, E) and *crk10-A397T* (B, D, F) plants. Stain: potassium permanganate. Scale bars: 100 µm (A, B, C, E, F) and 50 µm (D). (G, H) Detail of xylem vessels in hypocotyls of 4-week-old WT (G) and *crk10-A397T* (H) mutant plants. Stain: potassium permanganate. Insert in top left corner of image shows original micrographs. Sale bars:, 50 µm (inset G) and 25 µm (inset H). (I, J) Detection of autofluorescence of lignin on resin-embedded cross sections of 4-week-old hypocotyls of WT (I) and *crk10-A397T* (J) plants. Scale bars: 10 µm. (K–T) Transverse cross sections of resin-embedded hypocotyls of WT (K, M, O, Q, S) and *crk10-A397T* (L, N, P, R, T) plants at 1, 2, 3, 4, and 5 weeks after sowing. Stain: potassium permanganate. Scale bars: 50 µm (K, L, M, Q, R), 100 µm (N, S, T), and 25 µm (O, P). (U, V) Transverse cross sections of resin-embedded hypocotyls of 5-week-old WT (U) and *crk10-A397T* (V) plants; arrows indicate span of xylem I (XI) and xylem II (XII) areas. Stain: potassium permanganate. Scale bars, 100 µm (U) and 50 µm (V).

Dwarfism in plants is often caused by defects in the vascular system. To investigate whether the vasculature of the ­*crk10-A397T* mutant develops normally, we prepared ­transverse cross sections of resin-embedded hypocotyl, root, and stem samples of 5-week-old plants. The sections were stained with potassium permanganate, a lignin-specific dye that allows the observation of lignified xylem vessels and fibres. Imaging of the cross sections revealed that xylem vessels in the root and hypocotyl of the mutant plants were severely collapsed (Fig. 2C–F), whereas vessels in the stem remained largely unaffected (Fig. 2A, B), as confirmed additionally by Maeule staining (Supplementary Fig. S4A, B). To understand the progression of the phenotype, a developmental time series of hypocotyl cross sections spanning weeks 1–5 after sowing was analysed (Fig. 2K–V). Cross sections of 1- and 2-week-old hypocotyls revealed disorganization of xylem vessels in *crk10-A397T* plants at an early developmental stage as they did not proceed to form the typical radial patterning observed in the hypocotyl vasculature of WT plants (Fig. 2K–N). At 3 weeks of age, the first deformed and collapsed xylem vessels became apparent in the mutant hypocotyls, a phenotype that is even more severe in 4-week-old plants (Fig 2O–R). At 5 weeks of age, following the onset of flowering, cross sections revealed the absence of fully differentiated xylem fibres in the hypocotyl of the *crk10-A397T* plants, in contrast to the WT (Fig. 2S–V). Differentiation of xylary fibres in Arabidopsis hypocotyls is associated with the switch to growth phase II of xylem development, which is triggered by the transition to flowering. We conclude that this switch is delayed in the *crk10-A397T* plants, despite flowering occurring simultaneously to WT plants.

Interestingly, the collapsed xylem vessels displayed darker brown staining in response to the dye compared with their WT counterparts, which suggests that their secondary cell walls were more heavily lignified (Fig. 2G, H). This hypothesis was reinforced by detecting the auto-fluorescence of lignin of these cells using confocal microscopy, as the auto-fluorescence of xylem vessels in the mutant hypocotyl was consistently more intense than the signal obtained from WT (Fig. 2I, J).

### *CRK10* is expressed in close association with vascular tissues and the protein localizes to the plasma membrane

**Fig. 3.**
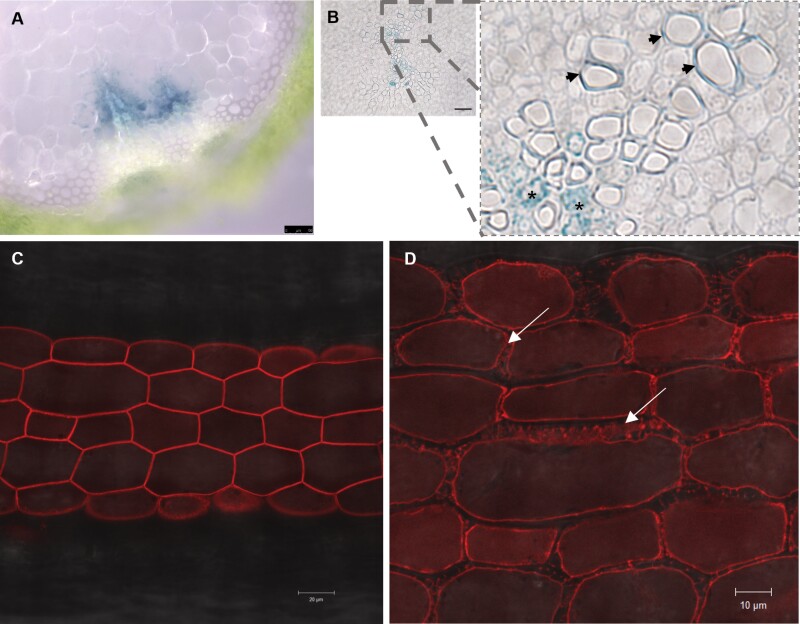
CRK10 is a plasma membrane-localized protein expressed in association with the vasculature in the hypocotyl. (A, B) Histochemical staining of reporter lines expressing the *CRK10*_Pro_:*GUS* construct showed expression of the reporter gene in the vasculature of stem and hypocotyls, as shown by free-hand cross section of 8-week-old inflorescence stem (A) and cross section of 2-week-old hypocotyl embedded in resin (B). Detail of cross section in (B) shows presence of histochemical staining in xylem parenchyma cells (asterisks) and differentiating xylem vessels (arrows) in the hypocotyl. Scale bars: 50 µm (A) and 25 µm (B). (C, D) Hypocotyl of 4-day-old seedling of transgenic Arabidopsis plant expressing 35S:*CRK10-mCherry* before (C) and after (D) plasmolysis. Representative Hechtian strands are indicated by white arrows. Scale bars: 20 µm.

Tissue-specific expression of *CRK10* was determined by placing the reporter *β-glucuronidase* under the control of the 1 kb genomic sequence containing the putative promoter of *CRK10* (*CRK10*_Pro_:*GUS*). *GUS* expression was detected in the vasculature of the roots, cotyledons, petioles, leaves, hypocotyls, and inflorescence stem (Fig. 3A, B; Supplementary Fig. S5A–D). In hypocotyls of 2-week-old seedlings, expression was localized to differentiating xylem vessels and parenchyma cells surrounding the vessel elements (Fig. 3B). The presence of the *CRK10* transcript in hypocotyls and inflorescence stems of 3- and 6-week-old WT and *crk10-A397T* mutant plants, respectively, was confirmed by qPCR (Supplementary Fig. S5E).

Subcellular localization of CRK10 was determined by analysing lines expressing a construct carrying the C-terminal translational fusion of *CRK10* with the fluorescent protein mCherry under the control of the constitutive 35S promoter (*35S*:*CRK10*-*mCherry*). Both transient expression of the construct in *N. benthamiana* leaves and stable expression in transgenic Arabidopsis plants indicated that the fusion protein localized to the plasma membrane (Supplementary Fig. S5F; Fig. 3C). The presence of Hechtian strands, characteristic of the retracting plasma membrane from the cell wall following plasmolysis (Oparka, 1994), further confirmed this subcellular localization of the protein (Fig. 3D). In order to exclude a possible effect of the point mutation on the subcellular localization of the protein, we also expressed the *35S:CRK10*^*A397T*^*-mCherry* variant transiently in tobacco leaves. Similar to the native protein, CRK10^A397T^-*mCherry* was found to localize to the plasma membrane, which suggests that the point mutation does not alter the subcellular localization of the protein and does not seem to affect its endocytosis (Supplementary Fig. S5G). Therefore, we conclude that *CRK10* is expressed in close association with vascular tissues of below- and above -ground organs, and that the protein localizes to the plasma membrane of plant cells.

### Collapsed xylem vessels in the root and hypocotyl are responsible for the dwarf phenotype of the *crk10-A397T* mutant

**Fig. 4.**
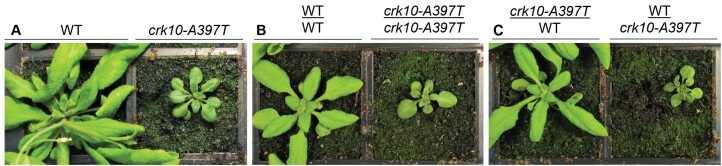
The root–hypocotyl system is responsible for the dwarf phenotype of *crk10-A397T* mutant plants. Images of non-grafted plants (A), self-graft controls (B), and graft combinations (C) of WT and *crk10-A397T* mutant. Plants were imaged 3 weeks after micrografting was performed. The phenotype observed for the reciprocal grafting combinations was consistently observed in two independent repetitions of the experiment. An average number of 10 grafts per combination was recovered each time. Annotation: scion/rootstock.

Although *CRK10* is expressed in tissues associated with vasculature in the stem, hypocotyl, and roots, as demonstrated by C*RK10*_Pro_:*GUS* analysis, it is intriguing that in the *crk10-A397T* plants xylem vessel collapse occurs only in roots and hypocotyls. To investigate if the dwarf phenotype is solely due to the defects of the belowground tissues, or whether it is a ‘whole-plant’ response, we performed a micrografting experiment (Turnbull *et al.*, 2002). *In vitro*-grown 4-day-old seedlings were used to generate combinations of WT rootstocks and *crk10-A397T* scions (WT/*crk10-A397T*) and vice versa (*crk10-A397T*/WT; Fig. 4C), as well as self-grafted plants as controls (WT/WT and *crk10-A397T*/*crk10-A397T*; Fig. 4B). Phenotypic assessment of successful grafts revealed that a WT scion grafted onto a *crk10-A397T* rootstock developed the characteristic dwarf phenotype of the mutant, whereas a ­mutant scion developed into a WT-like plant when grafted onto a WT rootstock (Fig. 4C). Our observations show that the root and hypocotyl system of the *crk10-A397T* plants are responsible for their dwarf phenotype, which is likely due to the presence of collapsed xylem vessels in these tissues.

### Loss of function or overexpression of *CRK10* does not have a phenotypic effect in Arabidopsis

In order to investigate whether increased levels of *CRK10* expression have phenotypic effects we introduced a construct carrying the WT cDNA of *CRK10* under the control of the constitutive 35S promoter in WT Arabidopsis plants. Two independent homozygous lines were generated and selected for further analysis (*CRK10* OE-1 and OE-2). Compared with WT, qPCR performed on 4-week-old leaves detected a *CRK10* transcript increase of 15 and 6 times for *CRK10* OE-1 and OE-2, respectively (Supplementary Fig. S6B), although growth and development were not altered (Supplementary Fig. S6A). In order to investigate whether the absence of the *CRK10* transcript affects the phenotype of Arabidopsis plants, two homozygous T-DNA knockout lines for the *CRK10* gene were isolated, *crk10-2* (SAIL_427_E09) and *crk10-4* (SALK_116653). Quantification of *CRK10* transcript levels from leaves of 4-week-old plants by qPCR confirmed that *crk10-2* and *crk10-4* are a knockout and knockdown line of *CRK10*, respectively (Supplementary Fig. S7B), but growth and development of both lines were indistinguishable from WT plants (Supplementary Fig. S7A). Cross sections of hypocotyls of 4-week-old *crk10-2* and *CRK10* OE-1 plants were imaged and showed that xylem vessels develop normally in both lines (Supplementary Fig. S8A–D).

### The A397T substitution is localized in the αC helix of the kinase domain of CRK10

**Fig. 5.**
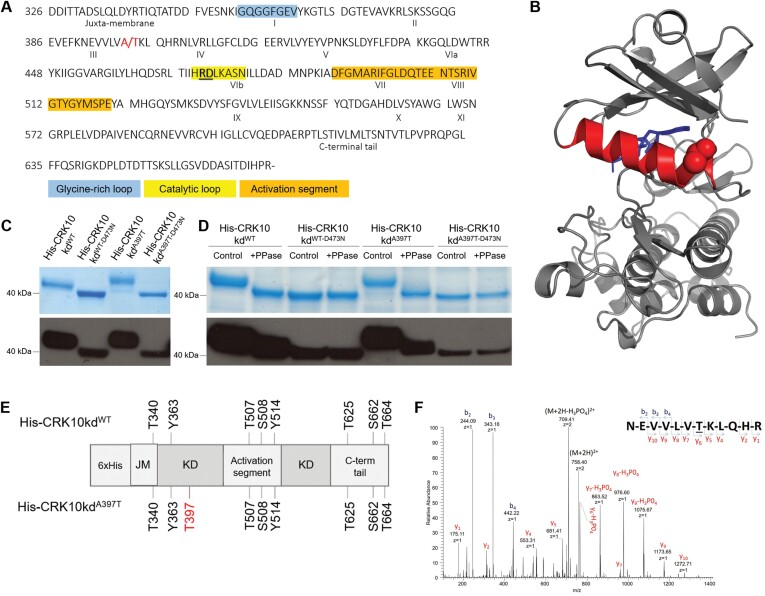
His-CRK10kd^WT^ and His-CRK10kd^A397T^ are active kinases and the A397T substitution introduces an additional autophosphorylation site in the kinase domain of the protein. (A) Amino acid sequence of the cytoplasmic kinase domain of CRK10 used for *in situ* phosphorylation studies (CRK10 amino acid residue numbering shown on the left). Subdomains I–XI are indicated by roman numerals. Ala/Thr397 site is highlighted in red, and the RD motif is underlined and highlighted in bold. The conserved glycine-rich loop, catalytic loop and activation segment motifs are also shown. (B) Structure of the CRK10 kinase domain generated by homology modelling to the active kinase domain of BRI1. A molecule of an ATP analogue, coloured blue, occupies the active site. The αC helix is coloured red, and the atoms in the side chain of the mutated threonine residue are depicted as red spheres. (C) Recombinant His-CRK10kd^WT^ and His-CRK10kd^A397T^ extracts and their respective dead kinase controls (His-CRK10kd^WT-D473N^ and His-CRK10kd^A397T-D473N^) resolved by SDS-PAGE; detection by western blot with horseradish peroxidase-conjugated anti-His antibody is shown. (D) SDS-PAGE and western blot following treatment of purified His-tagged proteins with λ-phosphatase (PPase); control: untreated protein. (E) *In situ* autophosphorylation sites in His-CRK10kd^WT^ and His-CRK10kd^A397T^ identified by LC-MS/MS analysis of the recombinant protein kinase domain. Threonine 397 is highlighted in red. C-term tail, C-terminal tail; His, 6× His-tag; JM, juxta-membrane domain; KD, kinase domain. (F) MS/MS spectrum of the doubly charged (*m*/*z* 758.4) tryptic phosphopeptide NEVVLVTKLQHR in which the threonine residue is phosphorylated. Neutral losses of phosphoric acid from both the precursor ion and the C-terminal y-ions are observed.

According to the subdivision of eukaryotic kinase domains into 12 conserved subdomains (Hanks and Hunter, 1995), the A397T substitution is localized in subdomain III of the kinase domain of CRK10 (Fig. 5A), which corresponds to the αC helix motif in the three-dimensional structure of the protein. Homology modelling to the active kinase domain of the Arabidopsis BRASSINOSTEROID INSENSITIVE 1 (BRI1) positions Thr397 at the C-terminal end of the helix, with its side chain likely to be exposed on the surface of the protein (Fig. 5B).

### The cytoplasmic kinase domain of CRK10 and *crk10-A397T* are active kinases

With CRKs being classified as Ser/Thr kinases, the replacement of Ala397 by a threonine (A397T) in the CRK10 kinase domain could have introduced a potential additional phosphorylation site. We therefore wanted to determine whether WT and mutant CRK10 are enzymatically active kinases and if differences in their autophosphorylation pattern could be detected. We addressed this question by investigating the autophosphorylation activity of the cytoplasmic kinase domain of CRK10 *in situ* when expressed in *E. coli* cells (Taylor *et al.*, 2013). We purified the CRK10 WT cytoplasmic kinase domain as an N-terminal 6× His-tag fusion protein (His-CRK10kd^WT^) from *E. coli* cells, as well as its ‘dead’ kinase counterpart that harboured the substitution of the essential aspartic acid 473 with an asparagine residue (His-CRK10kd^WT-D473N^). Following separation of the recombinant proteins by SDS-PAGE and detection by anti-His immunoblotting, the dead kinase version His-CRK10kd^WT-D473N^ migrated at the predicted molecular mass of 40 kDa, while the WT kinase version His-CRK10kd^WT^ showed an electrophoretic mobility shift to a larger molecular mass, known to occur for phosphorylated proteins (Wegener and Jones, 1984; Fig. 5C). In order to determine whether the A397T substitution in the CRK10 kinase domain affects its autophosphorylation activity, we generated constructs in which Ala397 was replaced by threonine through *in vitro* mutagenesis (His-CRK10kd^A397T^ and His-CRK10kd^A397T-D473N^). Although the dead kinase version His-CRK10kd^A397T-D473N^ migrated at the same molecular mass as His-CRK10kd^WT-D473N^ on SDS-PAGE gels, the mobility shift of His-CRK10kd^A397T^ was increased when compared with the one observed for His-CRK10kd^WT^ (Fig. 5C), suggesting additional sites were phosphorylated in His-CRK10kd^A397T^. In order to confirm that the electrophoretic mobility shift was due to the presence of phosphorylated residues, the purified recombinant proteins were treated with λ-phosphatase prior to separation by SDS-PAGE. Irrespective of the treatments, the dead kinase versions CRK10kd^WT-D473N^ and His-CRK10kd^A397T-D473N^ migrated at the predicted molecular mass as confirmed by SDS-PAGE and anti-His immunoblotting (Fig. 5D). However, λ-phosphatase treatment of His-CRK10kd^WT^ and His-CRK10kd^A397T^ resulted in a clearly detectable shift to a lower molecular mass, consistent with the autophosphorylation of recombinant His-CRK10kd^WT^ and His-CRK10kd^A397T^ as being responsible for their electrophoretic mobility shift. Taken together, these results confirm that both His-CRK10kd^WT^ and His-CRK10kd^A397T^ are active kinases capable of autophosphorylation. Furthermore, the increased mobility shift of His-CRK10kd^A397T^ compared with His-CRK10kd^WT^ suggested the presence of additional phosphorylation sites in His-CRK10kd^A397T^.

### The kinase domain of CRK10 autophosphorylates highly conserved residues in the activation loop and Thr397 is an additional phosphorylation site in His-CRK10kd^A397T^

We next proceeded to identify which sites in the kinase domain of CRK10 were being phosphorylated by subjecting tryptic peptides of His-CRK10kd^WT^ and His-CRK10kd^A397T^ to analysis by LC-MS/MS. The Mascot probability-based algorithm was used to confirm the peptides match to the CRK10 kinase domain sequence. Individual MS/MS spectra were inspected for confirmation of phosphorylation sites, which led to the unambiguous identification of Thr340, Tyr363, Thr507, Ser508, Tyr514, Thr625, Ser662, and Thr664 as phosphosites in both His-CRK10kd^WT^ and His-CRK10kd^A397T^ proteins (Fig. 5E). Interestingly, Thr507, Ser508, and Tyr514 align to conserved phosphorylation sites in the activation loop of several RLKs, known to be essential for the activation of RD kinases (Supplementary Fig. S9). Phosphorylated residues were also detected in the juxta-membrane region (Thr340) as well as in the C-terminal tail of CRK10 (Thr625, Ser662, and Thr664), which are predicted to act as regulatory sites for interaction with binding partners. In addition, the identification of two phosphorylated tyrosine residues (Tyr363 and Tyr514) classifies CRK10 as a dual specificity kinase and constitutes the first instance in which such activity has been reported for a CRK. Interestingly, Thr397 itself was identified as a phosphorylation site in the His-CRK10kd^A397T^ kinase domain *in situ* (Fig. 5F), but whether this residue also acts as a phosphorylation site *in vivo* remains to be determined.

### The hypocotyl transcriptome of *crk10-A397T* carries the signature of a plant responding to stress

**Fig. 6.**
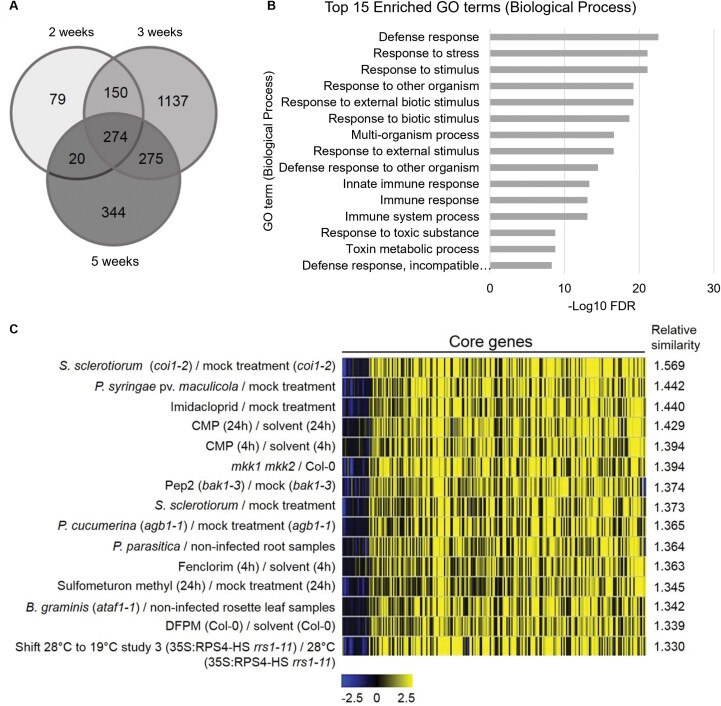
Transcriptional reprogramming in the *crk10-A397T* mutant shows activation of defence responses to biotic and abiotic stresses. (A) Venn diagram displaying the number of differentially expressed genes (DEGs) in the *crk10-A397T* hypocotyls compared with the WT at each developmental time point. (B) Top 15 enriched GO terms (Biological Process) for the up-regulated core DEGs in the *crk10-A397T* mutant plotted against their respective –log_10_ false discovery rate (FDR). (C) Top 15 perturbations showing highest overall similarity to *crk10-A397T* mutant expression signature (analysis performed using the GENEVESTIGATOR Signature tool; log_2_ fold change values of time point at 3 weeks after sowing was used as input for 274 core genes).

As the dwarf phenotype of the *crk10-A397T* mutant is associated with the collapse of xylem vessels in the belowground organs, we chose to investigate the effect of this mutation on the transcriptome of hypocotyls isolated from 2-, 3- and 5-week-old WT and mutant plants. Principal component analysis showed good clustering of replicates according to genotypes and developmental time points (Supplementary Fig. S10A). Following normalization and statistical analysis of the sequencing results (*q*≤0.05; log_2_ fold change threshold of ±1), we obtained 523 (2 weeks), 1836 (3 weeks), and 913 (5 weeks) DEGs, of which 274 were common to all time points. These DEGs were selected as the core set and taken forward for analysis (Fig. 6A; Supplementary Fig. S10B; Supplementary Tables S2–S5). Comparison with public datasets using the GENEVESTIGATOR Signature tool (Hruz *et al.*, 2008) revealed that the transcriptome signature of the *crk10-A397T* mutant was most similar to Arabidopsis plants challenged by fungal and bacterial pathogens (S*clerotinia sclerotorum*, *Plectosphaerella cucumerina*, and *Pseudomonas syringae*) and exposed to abiotic stresses (treatment with fenclorim and sulfometuron methyl) (Fig. 6C). Equally, Gene Ontology (GO) enrichment analysis of the up-regulated genes within the core set (246 genes) with AgriGO v.20 (Tian *et al.*, 2017; false discovery rate <0.05) revealed that terms associated with the biological functions ‘Defence response’ (GO:0006952; *P*=2.30 × 10^−26^), ‘Response to stimulus’ (GO:0050896; *P*=1.40 × 10^−24^) and ‘Response to stress’ (GO:0006950; *P*=2.00 × 10^−24^) are significantly over-represented (Fig. 6B; Supplementary Tables S6, S7). In accordance with the whole transcriptome data, marker genes indicative for the activation of the SA- and jasmonic acid (JA)-regulated defence pathways, such as pathogenesis-related and camalexin biosynthesic genes (Uknes *et al.*, 1992; Thomma *et al.*, 1998; Ahuja *et al.*, 2012) are significantly up-regulated in the *crk10-A397T* mutant (Supplementary Table S8). Transcription factors belonging to the WRKY, MYB, and NAC-domain containing families are prominent among the regulatory genes induced by *crk10-A397T*, many of which have been associated with the modulation of stress responses (Supplementary Table S9). The analysis of DEGs of individual time points, especially at 3 weeks after sowing, revealed the up-regulation of genes involved in the biosynthesis, signalling, and homeostasis of the hormone abscisic acid (ABA) (Supplementary Table S10). Also at this time point numerous cell wall-related genes were found to be differentially expressed in the mutant transcriptome, implying that widespread changes in cell wall composition and assembly could be responsible for the xylem vessel collapse observed in mutant hypocotyls (Supplemental Table S11). In summary, biotic and abiotic stress-responsive pathways are constitutively up-regulated in the *crk10-A397T* mutant, suggesting that *crk10-A397T* is a gain-of-function allele of *CRK10*.

### Differences in cell wall composition between wild type and mutant hypocotyls are manifold and complex

Xylem vessel collapse has been shown to be a consequence of altered cell wall composition leading to defective cell walls that are no longer able to withstand the negative pressure generated by transpiration, as for example described by Turner and Somerville (1997) for the *irregular xylem* (*irx*) mutants. As our transcriptomic dataset revealed the reprogramming of numerous cell wall-related genes, we proceeded with the characterization of the cell wall composition of the intact and collapsed xylem vessels in the hypocotyls of 3-week-old WT and *crk10-A397T* mutant plants by using FTIR spectroscopy, a powerful and rapid technique for analysing cell wall components and putative cross-links. The difference spectrum between the cell wall of an intact and collapsed xylem vessel revealed that complex changes had occurred in the collapsed vessel cell walls, with changes in hemicellulose composition and a reduced amount of ester cross-links among the most noticeable (Supplementary Fig. S11A). We further performed FTIR microscopy on transverse cross sections of 3-week-old hypocotyls of WT and mutant in order to map the differences in chemical composition onto the anatomical structure (Supplementary Fig. S11B–F). Heatmaps showed that the polysaccharide content was reduced in the cross sections in areas containing xylem elements in the mutant, and that the hemicellulose/cellulose ratio was altered (Supplementary Fig. S11C, D) as was the content of ester groups (Supplementary Fig. S11F). The complexity of the cell wall changes in the mutant, hinted at by FTIR, was further confirmed by quantification of total monosaccharides in the hypocotyls of 3-week-old WT and *crk10-mutant* plants (Supplementary Fig. S12). The content of multiple monosaccharides was changed in the mutant, with fucose, arabinose, and galactose showing a highly significant reduction, and xylose a highly significant increase.

In numerous mutants with collapsed xylem vessels, alteration of the cell wall composition can frequently be visualized as changes in the ultrastructure of the cell wall when analysed by transmission electron microscopy, as reported for the *irx1*, *irx3 irx8*, *irx7*, and *irx9* mutants (Taylor *et al.*, 2000; Persson *et al.*, 2007; Brown *et al.*, 2011), for example. However, contrary to our expectations, transmission electron microscopy analysis performed on the secondary cell walls of intact WT vessels and collapsed xylem vessels of the *crk10-A397T* mutant showed no obvious differences in the ultrastructure of the secondary cell walls (Supplementary Fig. S13) suggesting that the biochemical alterations in cell wall composition are not reflected by obvious structural defects that could explain the collapse of these cells. In summary, our cell wall analyses showed that many components of the vessel cell walls in the mutant are altered, in accordance with the transcriptional reprogramming suggested by our RNA-Seq experiment.

### *crk10-A397T* mutant hypocotyls contain increased levels of the stress hormones salicylic acid and abscisic acid

In order to corroborate the transcriptomic data, we quantified the levels of the stress hormones SA, ABA, and JA from the hypocotyls of 3-week-old WT and *crk10-A397T* plants (Supplementary Table S12). In agreement with the transcriptional induction of stress-responsive pathways, the levels of free SA and ABA were increased approximately 3 and 1.5 times, respectively, in the mutant hypocotyls. In contrast, JA levels were not significantly different to the WT.

### The *crk10-A397T* mutant displays enhanced resistance to infection by the vascular pathogen *Fusarium oxysporum* f. sp. *conglutinans* 699

**Fig. 7.**
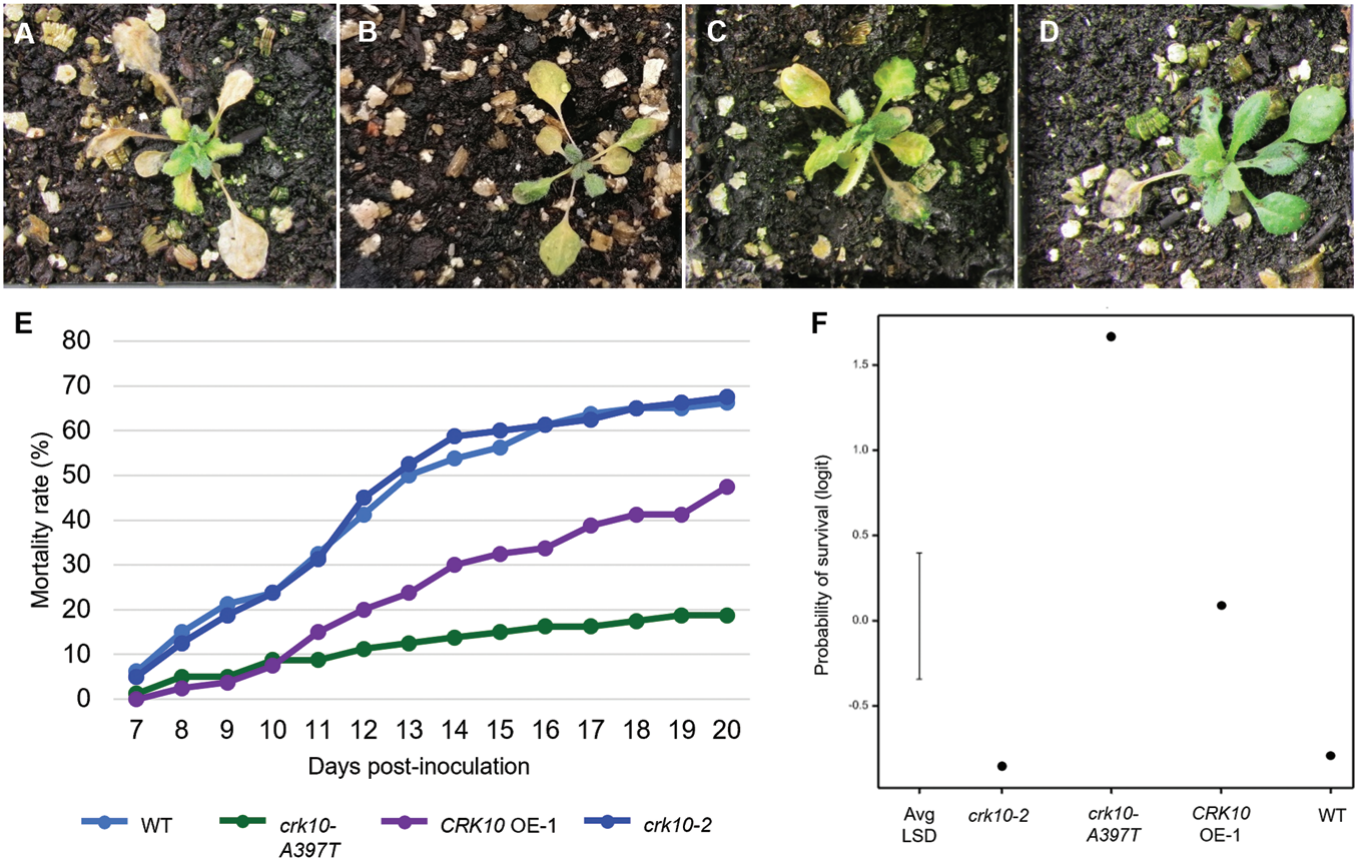
*crk10-A397T* mutant plants are more resistant to infection with *Fusarium oxysporum* f. sp. *conglutinans* 699. (A–D) Representative images of WT (A), *crk10-2* (B), *CRK10* OE-1 (C), and *crk10-A397T* mutant (D) plants at 11 d post-inoculation with *F. oxysporum*. (E) Mortality curve of WT, *crk10-A397T*, *CRK10* OE-1, and *crk10-2* plants from day 7 to 20 post-inoculation with *F. oxysporum*. Mortality is shown as a percentage. The experiment was repeated twice with similar results. (F) Probability of survival of each genotype following inoculation with the pathogen *F. oxysporum*. Associated 95% confidence intervals are shown. Deviance test, χ_3_^2^=19.68, *P*<0.001. LSD, least significant difference.

With defence responses constitutively up-regulated in the mutant, we next wanted to investigate whether this is reflected in an enhanced resistance to pathogens. Given that *CRK10* expression is detected mainly in the vasculature, we chose the vascular pathogen *Fusarium oxysporum* f. sp. *conglutinans* 699, an isolate known to infect Arabidopsis, for the assay (Masachis *et al.*, 2016). *CRK10* OE-1 and *crk10-2* lines were also included in the experiment as overexpression and knockout of other CRKs often showed enhanced/decreased resistance phenotype to pathogens (Acharya *et al.*, 2007; Yeh *et al.*, 2015; Yadeta *et al.*, 2017). The progression of the infection was observed (Fig. 7A–D) and a time–mortality curve was recorded from 7 to 20 d post-inoculation (Fig. 7E). Our results showed that the susceptibility of WT and *crk10-2* plants to the pathogens was very similar, with both genotypes reaching over 65% mortality at the end of the experiment. *CRK10* OE-1 and *crk10-A397T* plants exhibit a similarly low mortality rate until 10 d post-inoculation, although *CRK10* OE-1 plants reach a final death toll of 47.5%, in contrast to the lowest overall death count displayed by the *crk10-A397T* mutant of around 18%. Statistical analysis (deviance test, χ_3_^2^=19.68, *P*<0.001) confirmed the differences in the probability of survival between genotypes, with the *crk10-A397T* mutant having the highest chance of survival of 81.25%, followed by 52.5% for the *CRK10* OE-1 plants, and just over 30% for both *crk10-2* and WT (Fig. 7F). Fungal burden quantification by qPCR showed increased *F. oxysporum* biomass in WT and *crk10-2* plants compared with *CRK10* OE-1 and *crk10-A397T* mutant at 7 d post-inoculation, in agreement with the mortality trend results (Supplementary Fig. S14). The experiment was performed twice with similar results. Therefore, our results strongly suggest that *crk10-A397T* mutant plants are more resistant to infection with *F. oxysporum*, reinforcing our hypothesis that the transcriptional responses induced by the *crk10-A397T* mutant allele are effective at reducing the spread of root-infecting vascular pathogens.

## Discussion

Previous efforts to assign functions to specific CRKs mainly focused on the characterization of T-DNA knock-out and overexpression lines. However, with a large multigene family, the effects of knocking out one specific member are often masked by redundancy and, in the absence of known stimulants, lines constitutively overexpressing CRKs are frequently phenotypically indistinguishable from WT. Here we report the characterization of the *crk10-A397T* mutant that harbours a gain-of-function allele of *CRK10*, to our knowledge the first such mutant obtained for this class of receptors in Arabidopsis.

The point mutation responsible for the conversion of *CRK10* into a gain-of-function allele (alanine 397 to threonine) lies in subdomain III of the kinase domain, at the C-terminus of the αC helix and at the start of the short αC–β4 loop that links the αC helix to the β strand 4 (Fig. 5A). The importance of this region for kinase regulation has been studied in numerous mammalian kinases, with mutations residing in this area often leading to kinase deregulation and disease. Equivalent studies in plant kinases are, however, absent. The combined αC helix and αC–β4 loop have been shown to be critical allosteric docking sites that play a crucial part in kinase regulation (Yeung *et al.*, 2020). As the 3D model of the CRK10 kinase domain suggests, the substituted amino acid lies on that surface of the helix, which faces away from the active site of the kinase and that is known to provide an interface for interactions with regulatory domains and proteins (Fig. 5B). For human CDK2, for example, it was shown that residue Lys56, equivalent to the position of Thr397 in *crk10-A397T*, is involved in its interaction with cyclin necessary for the stabilization of the active form of the kinase (Jeffrey *et al.*, 1995), whereas in the case of the Ser/Thr-protein kinase B-Raf (BRaf), where dimerization is thought to be an important part of the activation mechanism, the αC helix/αC–β4 loop provides the interface for dimer formation (Rajakulendran *et al.*, 2009). This region can also function as a *cis*-regulatory site as shown for the human leucine-rich repeat kinase 2 (LRRK2), where it provides a firm docking site for the C-terminal residues of the kinase that keeps it in an inactive conformation (Deniston *et al.*, 2020; Taylor *et al.*, 2020). As the αC helix/αC–β4 loop is a feature common to all eukaryotic kinases, it seems reasonable to speculate that the amino acid substitution in *crk10-A397T* could perturb the interaction of CRK10 with a regulatory partner. It is noteworthy that the αC helix and αC–β4 loop is a highly conserved region within the family of CRKs, and only three residues are found to occupy the position equivalent to Ala397 of CRK10 among all the members: alanine, threonine, or serine (Supplementary Figs S15, S16). It now remains to be seen whether other members of the CRK family are similarly affected by an analogous mutation in their αC helix/αC–β4 loops. It also is notable that the only other semi-dominant mutant reported for a CRK in rice, *als1*, which develops spontaneous lesions on leaf blades and sheaths, harbours a point mutation that also localizes in the vicinity of the αC helix (Du *et al.*, 2019).

*In situ* phosphorylation analysis of the cytoplasmic kinase domains of WT and mutant CRK10 by LC-MS/MS determined that CRK10, being an RD kinase, shows the typical autophosphorylation pattern of conserved phosphorylation sites in the activation loop, on which the activity of this class of kinases depends (Fig. 5E; Nolen *et al.*, 2004; Beenstock *et al.*, 2016). Thr507 and Ser508 were identified as unambiguous phosphorylation sites, with Ser508 likely to be functionally equivalent to Thr450 of BAK1, which plays a key role in its activation by maintaining the active conformation of the activation loop (Yan *et al.*, 2012). However, we also detected phosphorylation of tyrosine residues (Tyr363 and Tyr514), with Tyr514 residing in the activation loop, which establishes CRK10 as a dual specificity kinase. Additional phosphorylated residues were detected in the juxtamembrane domain (Thr340) and at the C-terminus (Thr625, Ser662, and Thr664). Phosphorylation of residues in these non-catalytic regions has been shown to play an important role in the recognition and/or phosphorylation of downstream substrates, although these are unknown for CRKs (Wang *et al.*, 2005; Oh *et al.*, 2012; Zhou *et al.*, 2020). The phosphorylation pattern of His-CRK10kd^WT^ and His-CRK10kd^A397T^ was identical, with the notable exception of the substituted Thr397 functioning as an additional phosphorylation site in His-CRK10kd^A397T^ (Fig. 5E). Whether these phosphorylation sites, which were identified *in vitro*, do play a role *in vivo* and the determination of their biological significance are questions which need to be addressed in future work. For example, introducing a phosphomimic variant of threonine 397 *in planta* could ascertain that there is a link between the phosphorylation of threonine 397 and the *crk10-A397T* mutant phenotype. It will also be important to determine if/how the mutation affects kinase activity. As substrates phosphorylated by CRK10 remain unknown, artificial substrates such as myelin basic protein could be used in *in vitro* kinase assays in order to unravel whether the *crk10-A397T* phenotype can be linked to, for example, enhanced kinase activity. In addition, interacting partners of the WT and mutant variants of CRK10 *in vivo* could be identified, which will help to establish whether the A397T mutation perturbs and/or promotes protein–protein interactions, thus affecting the dynamics of the receptor complexes in which CRK10 resides.

Phenotypically the *crk10-A397T* mutant is a dwarf with severely collapsed xylem elements in the roots and hypocotyl, whereas xylem elements in the inflorescence stem remain intact (Figs 1, 2). A grafting experiment suggested that it is this defective vasculature in the belowground organs that causes the dwarf phenotype, as WT scions grafted onto *crk10-A397T* rootstocks become similarly stunted, whereas *crk10-A397T* scions grafted on WT rootstocks develop normally (Fig. 4). As *CRK10* is expressed in vascular-associated tissues in both stem and hypocotyl, as shown by reporter lines (Fig. 3A, B) and qPCR, a tissue-specific expression of *CRK10* cannot, therefore, explain why in the *crk10-A397T* mutant the xylem elements are defective in one organ but not the other. In the absence of detailed knowledge on how the mutation alters CRK10 function, an explanation for this observed discrepancy remains highly speculative. For example, one straightforward hypothesis could envisage that the alanine 397 substitution by threonine alters the substrate recognition domain and confers to it the ability to phosphorylate substrates that are present in the hypocotyl but absent in the stem. Alternatively, stem and hypocotyl might differ in the composition of the receptor complexes within which CRK10, in analogy to other receptor-like kinases, is anticipated to reside. As a result, the interaction with regulatory partners within the complex might be differently affected by the *crk10*-A3*97T* mutation leading, for example, to kinase activation in one tissue but not the other.

Collapsed xylem vessels are thought to be a consequence of alterations in cell wall composition, leading to cell wall weakening and an inability to withstand the negative pressure generated by transpiration, as reported, for example, in the *eskimo* and *irx* mutants (Turner and Somerville, 1997; Lefebvre *et al.*, 2011). The collapse of xylem vessels in the hypocotyl of the *crk10-A397T* mutant plants also seems to be accompanied by changes in cell wall composition, as suggested by the transcriptional reprogramming of multiple cell wall-related genes revealed in our transcriptomic dataset. Corroborating this finding, vessel cell wall analysis with FTIR and monosaccharide analysis of the hypocotyl revealed that changes occurring in the cell wall composition of the mutant were complex with several compounds being affected, amongst which hemicellulose and ester cross-links were the most prominent. The alteration in chemical composition, however, was not reflected in alterations of the ultrastructure of the vessel cell walls, as transmission electron microscopy analysis performed on cross sections of 3-week-old hypocotyls of the *crk10-A397T* mutant and wild-type was unable to detect any obvious differences. Preliminary analysis for the determination of the underlying cause of the vessel collapse remains therefore inconclusive and warrants a separate line of investigation that is beyond the scope of this work.

Collapsed vessel elements very likely impede water transport, which could be perceived as drought stress leading to an increase of ABA. This could explain the elevated levels of ABA that we detected in the hypocotyl of *crk10-A397T* and the up-regulation of genes involved in ABA synthesis, perception, and response (Supplementary Table S10). A similar increase in ABA levels has been observed in other cell wall mutants such as *esk1* and *irx1-6* whereas *irx3*, *irx5*, and *irx9* contained numerous constitutively up-regulated ABA-responsive genes (Chen *et al.*, 2005; Hernández-Blanco *et al.*, 2007; Lefebvre *et al.*, 2011; Faria-Blanc *et al.*, 2018; Xu *et al.*, 2020). The stunting of these mutants has been suggested to be the consequence of the response to drought signalling hormones resulting in the suppression of growth, which could also explain the dwarfism of *crk10-A397T*. It will be interesting to investigate whether dwarfing of *crk10-A397T* can be alleviated by an ABA-insensitive mutant such as *abi1* (Koornneef *et al.*, 1984).

Alteration of cell wall composition can lead not only to loss of cell wall rigidity, as it is increasingly being reported that modification of cell wall composition by genetic or chemical means leads to the constitutive activation of defence pathways and an altered resistance to pathogens. The primary cell wall mutant *ixr1/cev1*, for example, with reduced crystalline cellulose content due to the defective cellulose synthase CESA3, displays constitutive activation of JA and ethylene signalling (Ellis and Turner, 2001; Ellis *et al.*, 2002). Transcriptomic data obtained for the secondary cell wall mutants *irx1-6*, *irx5-5*, and *irx9* showed that defence-related genes are constitutively expressed in these mutants (Hernández-Blanco *et al.*, 2007; Faria-Blanc *et al.*, 2018). In line with these reports, our data showed that the signature of the core set of DEGs of *crk10-A397T* was most similar to the transcriptome of plants responding to biotic and abiotic stress (Fig. 6). Canonical SA-dependent marker genes (*PR1*, *PR2*, and *PR5*) and genes involved in the synthesis of the tryptophan-derived antimicrobial compounds (camalexin, glucosinolates) are significantly up-regulated in the mutant, as are numerous transcription factors usually associated with co-ordinating stress responses (Supplementary Tables S8, S9). Concomitant with gene induction, SA levels in the *crk10-A397T* mutant hypocotyls are increased, whereas changes in JA levels were not significant. Induction of defence pathways due to cell wall impairment manifests itself frequently in alteration of disease resistance to a wide range of pathogens (Houston *et al.*, 2016; Bacete *et al.*, 2018). Molina *et al.* (2021), for example, showed that from a panel of 34 cell wall mutants affecting a wide range of different cell wall compounds, 29 had an altered, mainly enhanced, resistance response to pathogens comprising different parasitic lifestyles. In order to assess disease resistance of *crk10-A397T* we chose the root-infecting, hemi-biotrophic vascular wilt pathogen *F. oxysporum* to perform a pathogen assay, bearing in mind the vasculature-associated expression of *CRK10*. In agreement with the studies linking cell wall modification to altered disease resistance, we found that the *crk10-A397T* mutant was significantly more resistant to the pathogen, part of which could be due to the fact that collapsed xylem vessels act as physical barriers slowing pathogen progression in the roots (Fig. 7).

Taken together, the experimental evidence provided in this report suggests that *crk10-A397T* is a gain-of-function allele of *CRK10* as activation of defence pathways occurs in the absence of a stimulus in the mutant. However, our observations do not currently allow us to infer the biological function of CRK10 as, according to Muller (1932), gain-of-function phenotypes can be the consequence of different types of mutations, namely neomorphic, hypermorphic, or antimorphic. As long as mode of action and regulation remain uncharacterized for CRKs, it is not possible to ascertain which type of gain-of-function mutation the *crk10-A397T* allele represents, which makes it difficult to determine the role of CRK10 *in planta*.

Another outstanding question in order to uncover the role of CRK10 remains the identification of its ligand. Intriguingly, plant DUF26 domains share strong structural similarity with fungal carbohydrate-binding modules, which led Vaattovaara *et al.* (2019) to propose that DUF26 proteins could be carbohydrate-binding domains, and cell-wall-derived carbohydrates or small extracellular molecules could represent candidate ligands. However, efforts to prove carbohydrate binding to the DUF26 domain have so far not been successful and bona fide ligands are still awaiting discovery.

## Supplementary data

The following supplementary data are available at *JXB* online. 

Fig. S1. *crk10-A397T* is a semi-dominant allele of *CRK10*.

Fig. S2. *crk10-A397T* mutant seeds germinate earlier than the WT and exhibit increased seedling size.

Fig. S3. Reduction in rosette size and aborted apical meristem of *crk10-A397T* plants.

Fig. S4. Xylem vessels in the stem of the *crk10-A397T* mutant do not collapse and lignification in the stem of WT and the *crk10- A397T* mutant is restricted to xylem vessels and fibres.

Fig. S5. *CRK10* is expressed in vascular tissues and the protein localizes to the plasma membrane.

Fig. S6. Transgenic plants overexpressing the *CRK10* transcript develop normally and resemble the WT.

Fig. S7. T-DNA knockout mutants of *CRK10* develop normally and resemble the WT.

Fig. S8. The hypocotyls of *crk10-2* and *CRK10* OE-1 plants do not contain collapsed xylem vessels.

Fig. S9. Alignment of the activation segment of eukaryotic kinase domains shows highly conserved residues and phosphorylation sites.

Fig. S10. Principal component analysis (PCA) plot of RNA sequencing samples and number of differentially expressed genes identified in *crk10-A397T* mutant hypocotyls.

Fig. S11. The FTIR spectrum profile of collapsed xylem vessels in the *crk10-A397T* mutant hypocotyls shows marked differences from the spectrum of intact vessels in the WT.

Fig. S12. The total monosaccharide content of hypocotyls of *crk10-A397T* mutant plants shows marked differences from that of WT plants.

Fig. S13. The ultrastructure of the secondary cell wall of collapsed xylem vessels resembles that of intact vessels in the WT.

Fig. S14. Fungal burden quantification at 2 and 7 d post-inoculation with *F. oxysporum*.

Fig. S15. Alignment of the αC helix segment of the kinase domain of the CRK family from Arabidopsis shows members of the family that contain alanine, threonine, or serine residues on position equivalent to Ala397 in CRK10.

Fig. S16. Phylogenetic tree of the CRK family from Arabidopsis and their respective residue on position equivalent to Ala397 in CRK10.

Table S1. Table of primers.

Tables S2–S5. Differentially expressed genes in the *crk10-A397T* mutant hypocotyls.

Tables S6, S7. Gene Ontology analysis.

Tables S8–S11. Tables of differentially expressed genes per functional category.

Table S12. Quantification of hormones in the hypocotyl of WT and *crk10-A397T* mutant plants.

erad080_supp_Supplementary_Figs_S1-S16_and_Tables_S1_S8-S12

erad080_suppl_Supplementary_Tables_S2-S7

## Data Availability

The data supporting the findings of this study are available from the corresponding author upon request. RNAseq data were uploaded to NCBI BioProject and can be accessed under https://www.ncbi.nlm.nih.gov/bioproject/901957. The mass spectrometry proteomics data have been deposited to the ProteomeXchange Consortium via the PRIDE (Perez-Riverol *et al.*, 2019) partner repository with the dataset identifier PXD023831.

## Abbreviations

CRK: cysteine-rich receptor-like kinase
RLK: receptor-like kinase
