# Supplementary material for: A point mutation in the kinase domain of CRK10 leads to xylem vessel collapse and activation of defence responses in Arabidopsis
Source: J Exp Bot. 2023 Mar 3;74(10):3104–21. doi: 10.1093/jxb/erad080 (PMC10199123; doi:10.1093/jxb/erad080)
Supplement: erad080_supp_Supplementary_Figs_S1-S16_and_Tables_S1_S8-S12 [file erad080_supp_supplementary_figs_s1-s16_and_tables_s1_s8-s12.pdf]

Supplementary Data

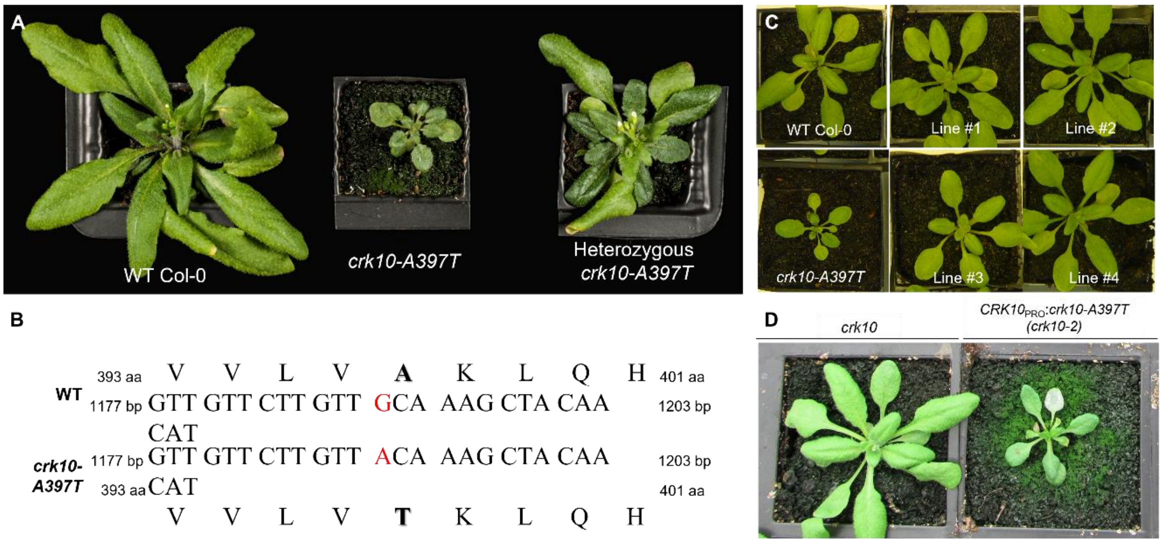

Supplementary Figure S1 *crk10-A397T* is a semi-dominant allele of *CRK10*.

(A) Segregating phenotypes of progeny from backcross between *crk10-A397T* mutant and WT Col-0.

(B) Single point mutation harboured by the *crk10-A397T* mutant is highlighted in red (G>A on position 1189 of coding sequence of *CRK10*) and associated amino acid substitution is shown (alanine > threonine on position 397 of the protein sequence of CRK10).

(C) WT Col-0 and *crk10-A397T* plants next to four independent complementation lines expressing the WT sequence of *CRK10* (35S:*CRK10*^{WT}) in the mutant background.

(D) *crk10-2* next to transgenic plant expressing the *crk10-A397T* allele driven by the *CRK10* native promoter (*CRK10*$_{Pro}$:*crk10-A397T*) in the *crk10-2* background.

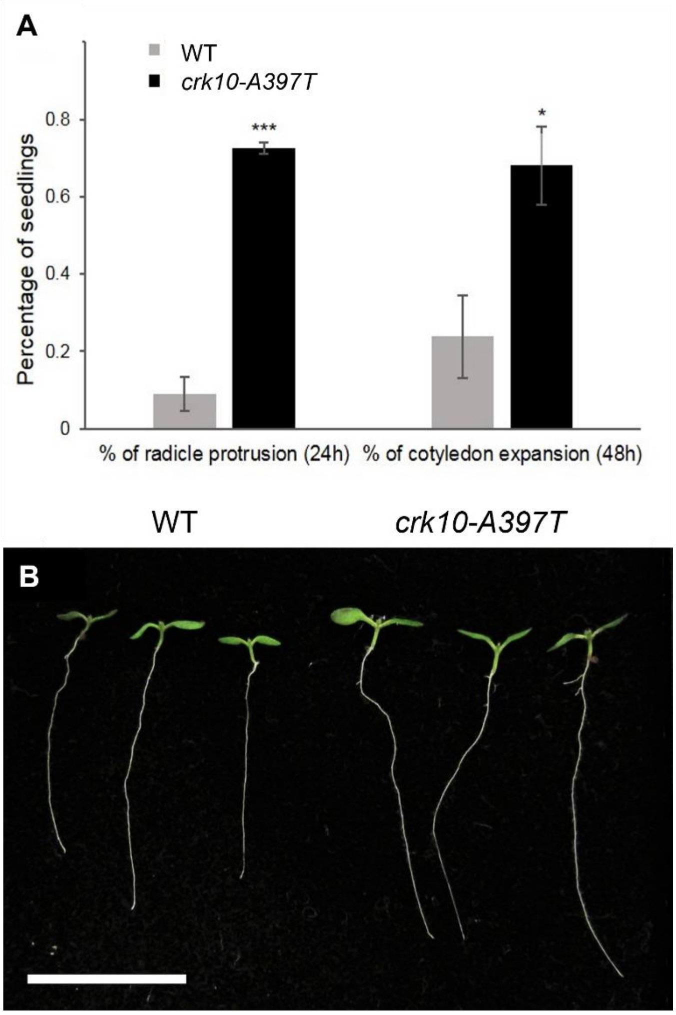

Supplementary Figure S2 *crk10-A397T* mutant seeds germinate earlier than the WT and exhibit increased seedling size.

(A) Germination rate of WT and *crk10-A397T* seeds after 24 and 48 hours of exposure to continuous light; germination was assessed on MS ½ agar plates following stratification for 48 hours. Error bars represent the standard error of three biological replicates (n=50). Asterisks indicate statistical significance (t-test): $^{*} = p \leq 0.05$; $^{***} = p \leq 0.001$.

(B) 4-day-old seedlings grown under continuous light. Bar, 1 cm.

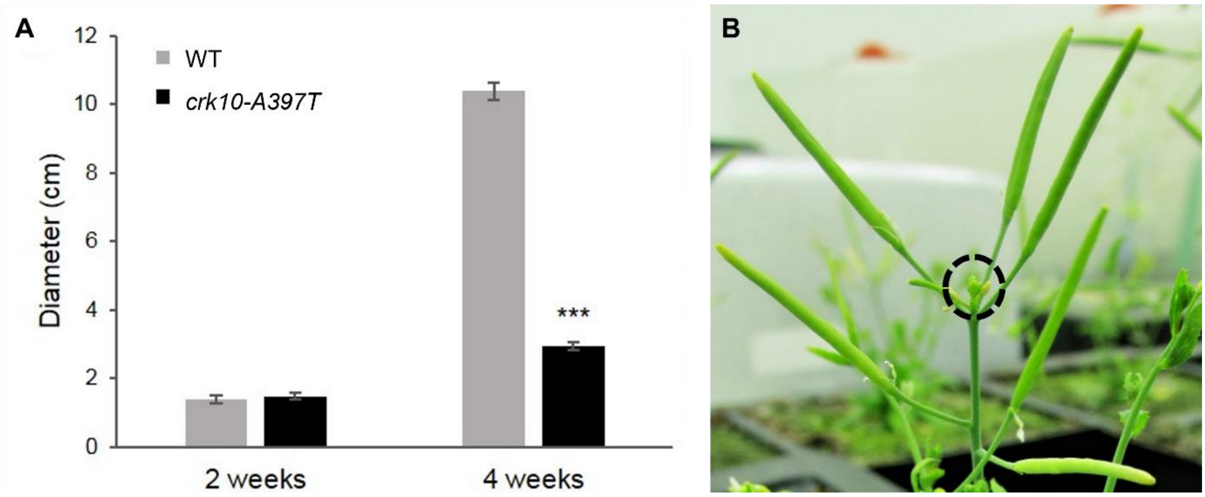

Supplementary Figure S3 Reduction in rosette size and aborted apical meristem of *crk10-A397T* plants.

(A) Average rosette diameter of WT and *crk10-A397T* plants at 2 and 4 weeks after sowing. Error bars represent the standard error of the mean (n = 10). Asterisks indicate statistical significance (t-test): $^{***} = p \leq 0.001$.

(B) Stunted inflorescence shoot of 6-week-old *crk10-A397T* plant. Dashed circle indicates aborted shoot apical meristem.

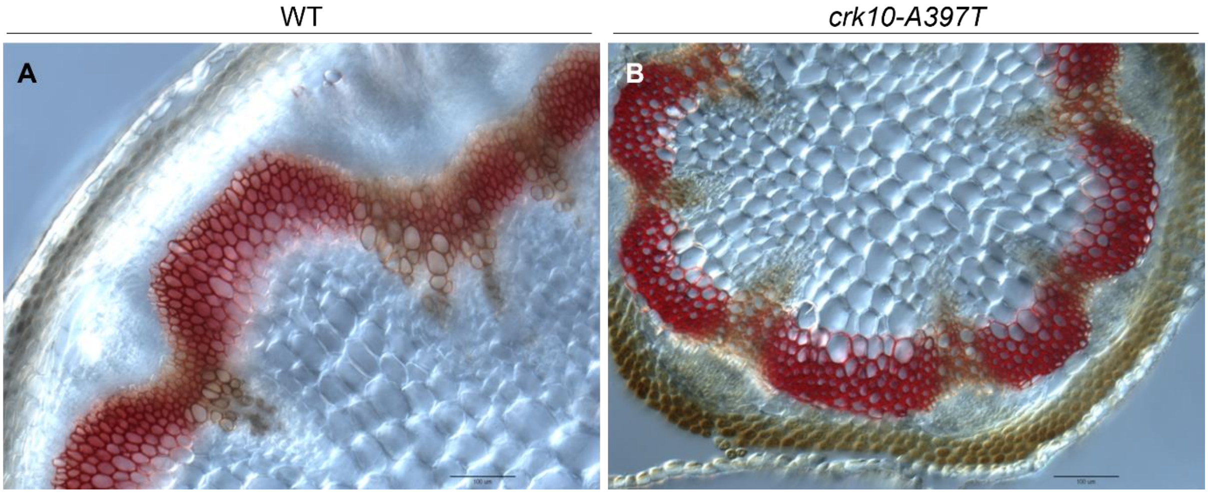

Supplementary Figure S4 Xylem vessels in the stem of the *crk10-A397T* mutant do not collapse and lignification in the stem of WT and the *crk10- A397T* mutant is restricted to xylem vessels and fibers.

(A-B) Hand cross sections were prepared from the base of the inflorescence stem of 7-week-old WT (A) and *crk10-A397T* mutant (B) plants and stained with Maeule. Asterisks indicate xylem vessels; arrowheads indicate fibers. Three biological replicates were analysed per genotype. Bars, 100 µm.

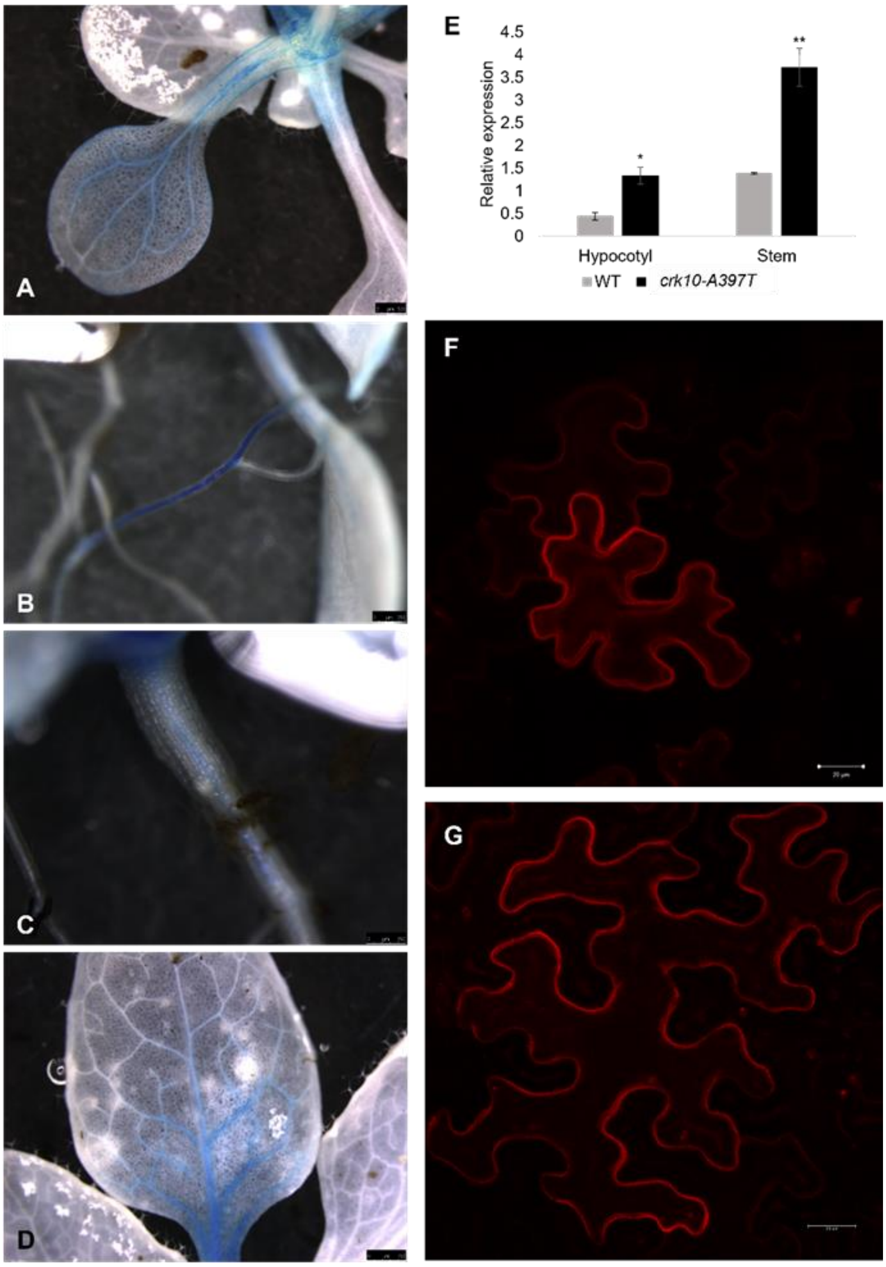

Supplementary Figure S5 *CRK10* is expressed in vascular tissues and the protein localises to the plasma membrane.

(A-D) Histochemical staining of reporter lines expressing the *CRK10*$_{\text{Pro}}$:*GUS* construct showed expression of the reporter gene in the vasculature of 2-week-old cotyledons and petioles (A), 10-day-old roots (B), 2-week-old hypocotyls (C) and 3-week-old leaves (D). Bars, (A) = 500 µm; (B, C) = 250 µm; (D) = 750 µm.

(E) Quantification of the relative expression of the *CRK10* transcript by qPCR. Template cDNA was synthesized from RNA extracted from hypocotyls of 3-week-old plants and inflorescence stem of 6-week-old plants; genes *AtACTIN2* and *AtUBC21* were used as internal controls. Error bars represent standard error of three biological replicates. Asterisks indicate statistical significance (t-test): * = $p \leq 0.05$; ** = $p \leq 0.01$.

(F-G) Cells from *Nicotiana benthamiana* leaf transiently expressing (F) *35S:CRK10-mCherry* and (G) *35S:CRK10*^{A397T}*-mCherry* constructs. Bars, 20 µm.

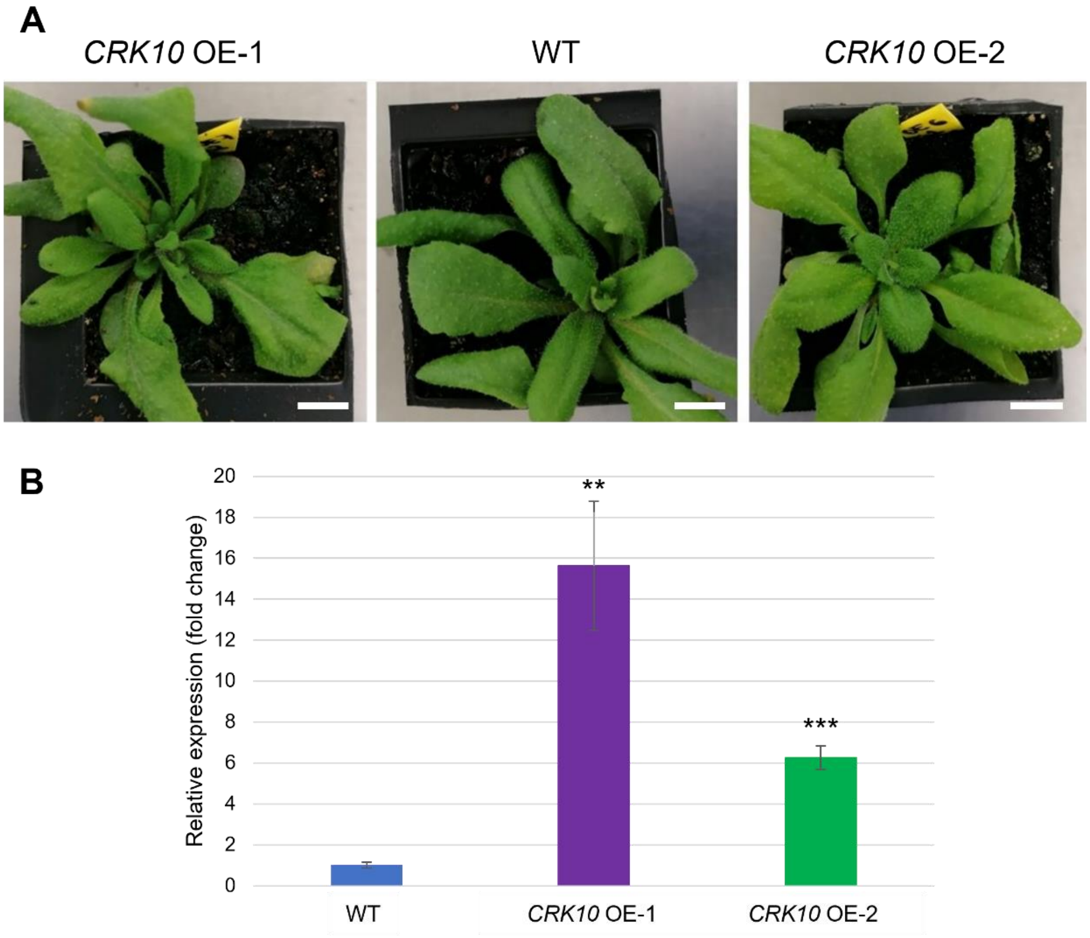

Supplementary Figure S6 Transgenic plants overexpressing the *CRK10* transcript develop normally and resemble the WT.

(A) Representative image of 4-week-old plants grown under standard long day conditions. Bars, 1 cm.

(B) Quantification of the relative expression of the *CRK10* transcript by qPCR. Template cDNA was synthesized from RNA extracted from leaves of 4-week-old plants; genes *AtACTIN2* and *AtUBC21* were used as internal controls. Error bars represent standard error of three biological replicates. Asterisks indicate statistical significance (t-test): $^{**} = p \leq 0.01$; $^{***} = p \leq 0.001$.

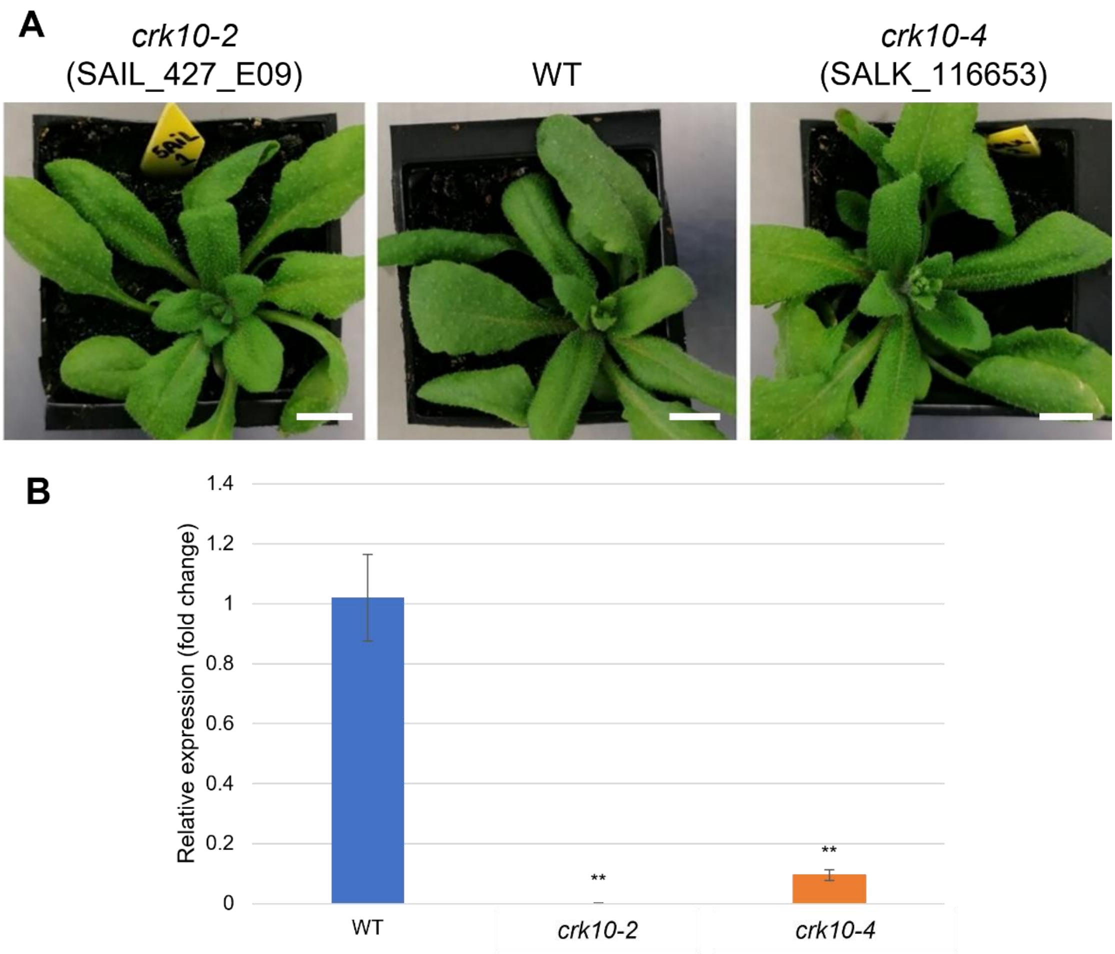

Supplementary Figure S7 T-DNA knockout mutants of *CRK10* develop normally and resemble the WT.

(A) Representative image of 4-week-old plants grown under standard long day conditions. Bars, 1 cm.

(B) Quantification of the relative expression of the *CRK10* transcript by qPCR. Template cDNA was synthesized from RNA extracted from leaves of 4-week-old plants; genes *AtACTIN2* and *AtUBC21* were used as internal controls. Error bars represent standard error of three biological replicates. Asterisks indicate statistical significance (t-test): ** = $p \leq 0.01$.

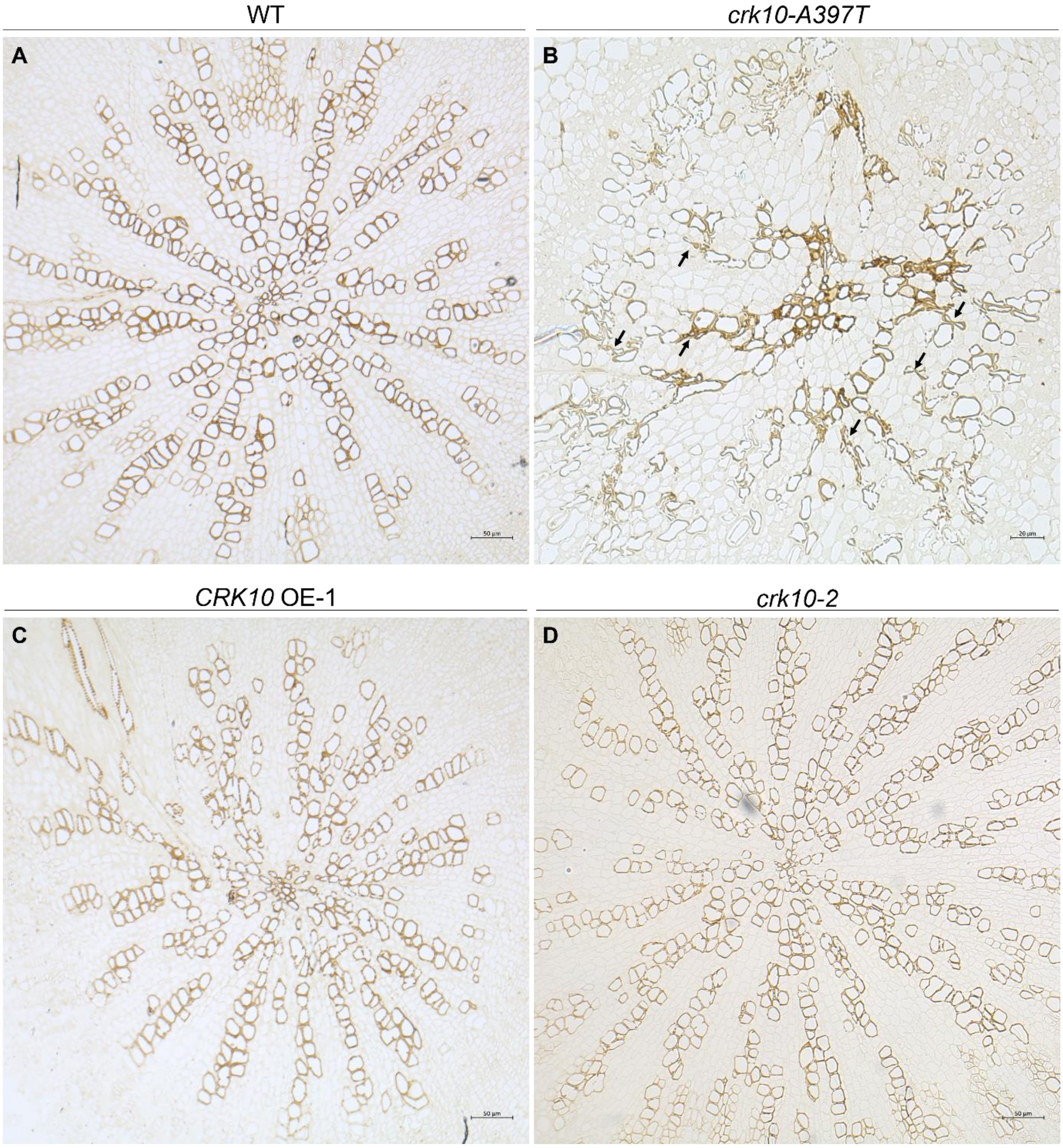

Supplementary Figure S8 The hypocotyls of *crk10-2* and *CRK10* OE-1 plants do not contain collapsed xylem vessels.

(A-D) Cross sections of resin embedded hypocotyls of 4-week-old WT (A), *crk10-A397T* (B), *CRK10* OE-1 (C) and *crk10-2* (D) plants. Stain: potassium permanganate. Black arrows indicate collapsed xylem vessels in the *crk10-A397T* mutant hypocotyl (B). Three biological replicates were analysed per genotype. Bars, (A, C, D) = 50 µm; (B) = 20 µm.

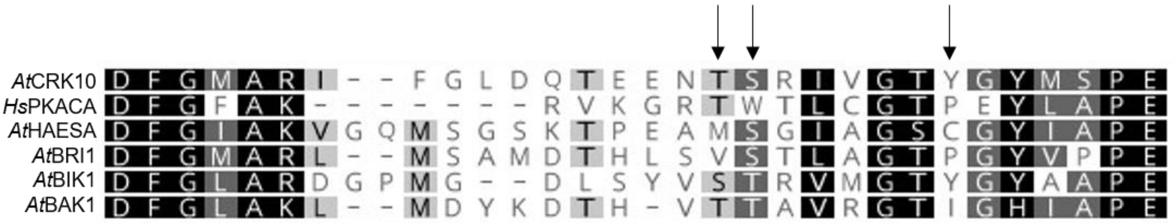

Supplementary Figure S9 Alignment of the activation segment of eukaryotic kinase domains shows highly conserved residues and phosphorylation sites. The activation segment of the kinase domain of CRK10, BAK1, BIK1, BRI1 and HAESA from *A. thaliana*, and PKACA from *Homo sapiens*, were imported from Uniprot (Uniprot Consortium; uniprot.org) and aligned using the Geneious Alignment tool (Geneious 10.0.9); alignment type: global alignment with free end gaps; cost matrix: Blosum62. Black arrows indicate position of phosphorylated residues Thr507, Ser508 and Tyr514 in the activation segment of CRK10. CRK10 Ser508 aligns to BAK1 Thr450, BIK1 Thr237, BRI1 Ser1044 and HAESA Ser856; CRK10 Tyr514 aligns to BIK1 Tyr243.

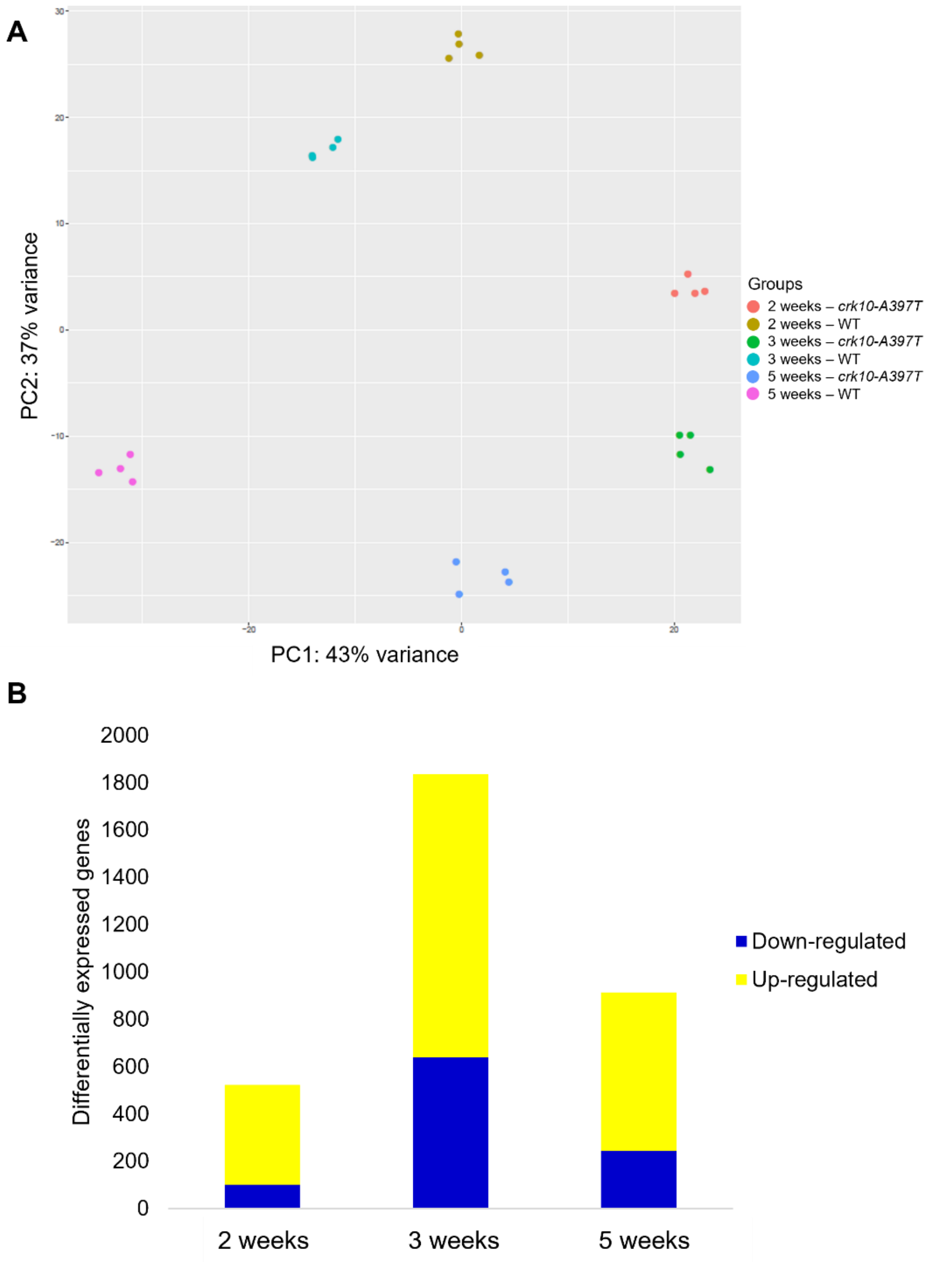

Supplementary Figure S10 Principal component analysis (PCA) plot of RNA sequencing samples and number of differentially expressed genes identified in *crk10-A397T* mutant hypocotyls.

(A) A principal components analysis (PCA) plot was generated using the PCA plot function in the Bioconductor package DESeq2, which plots rlog (https://rdrr.io/bioc/DESeq2/man/rlog.html) transformed data.

(B) Number of differentially expressed genes in the *crk10-A397T* mutant hypocotyls compared to the WT at each developmental time point.

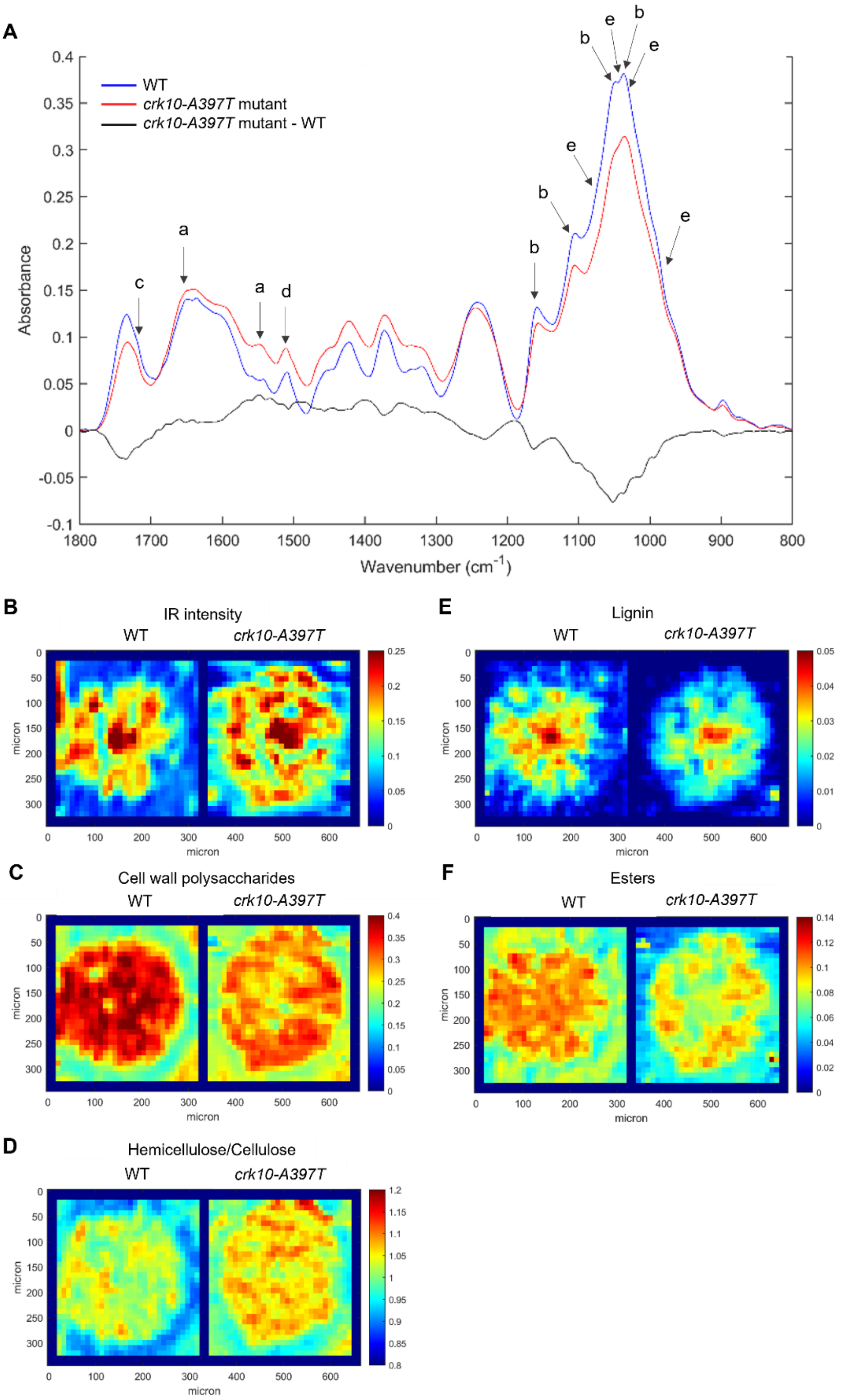

Supplementary Figure S11 The FTIR spectrum profile of collapsed xylem vessels in the *crk10-A397T* mutant hypocotyls shows marked differences to the spectrum of intact vessels in the WT.

(A) FTIR spectrum of intact xylem vessels (WT), collapsed xylem vessels (*crk10-A397T*), and the resulting difference spectrum (*crk10-A397T* – WT) are shown. The spectra are representative of the analysis of three biological replicates per genotype. Peaks corresponding to specific cell wall components are indicated by letters: (a) protein amide (1650 and 1544 cm^{-1}); (b) polysaccharides (1161, 1103, 1055 and 1036 cm^{-1}); (c) ester carbonyl (1733 cm^{-1}); (d) lignin phenolic groups (1507 cm^{-1}); (e) xyloglucan (1082, 1047, 1025 and 978 cm^{-1}).

(B-F) Representative FTIR chemical maps of cross sections of 3-week-old WT and *crk10-A397T* mutant hypocotyls. Maps show infrared (IR) intensity/spectral density (B), the polysaccharide band maximum (C), the hemicellulose/cellulose band ratio (1033 cm^{-1} /1050 cm^{-1}) (D), the lignin band (1510 cm^{-1}) intensity (E), and the ester band (1735 cm^{-1}) intensity (F). Maps were generated based on three biological replicates.

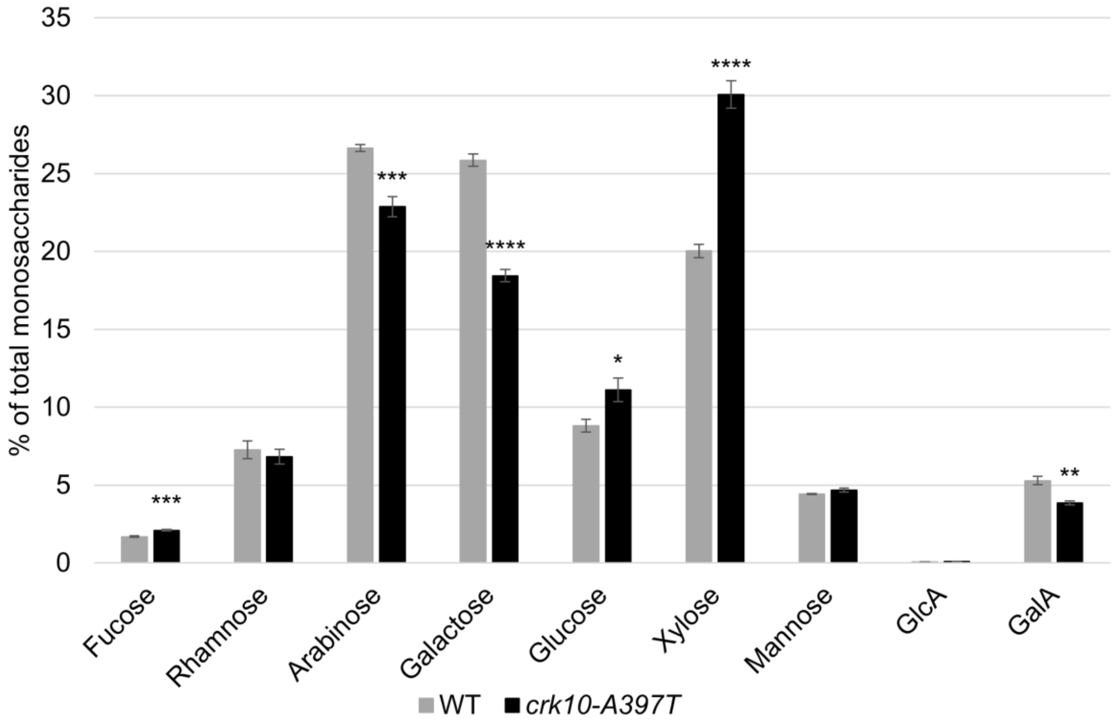

Supplementary Figure S12 The total monosaccharides content of hypocotyls of *crk10-A397T* mutant plants show marked differences to that of WT plants. The bar chart shows the relative content of neutral and acidic monosaccharides from the hypocotyls of 3-week-old WT and *crk10-A397T* mutant plants. Error bars represent the standard error of five biological replicates (each biological replicate was comprised of a pool of 30 hypocotyls). Asterisks indicate statistical significance (t-test): * = $p \leq 0.05$; ** = $p \leq 0.01$ *** = $p \leq 0.001$; **** = $p \leq 0.0001$

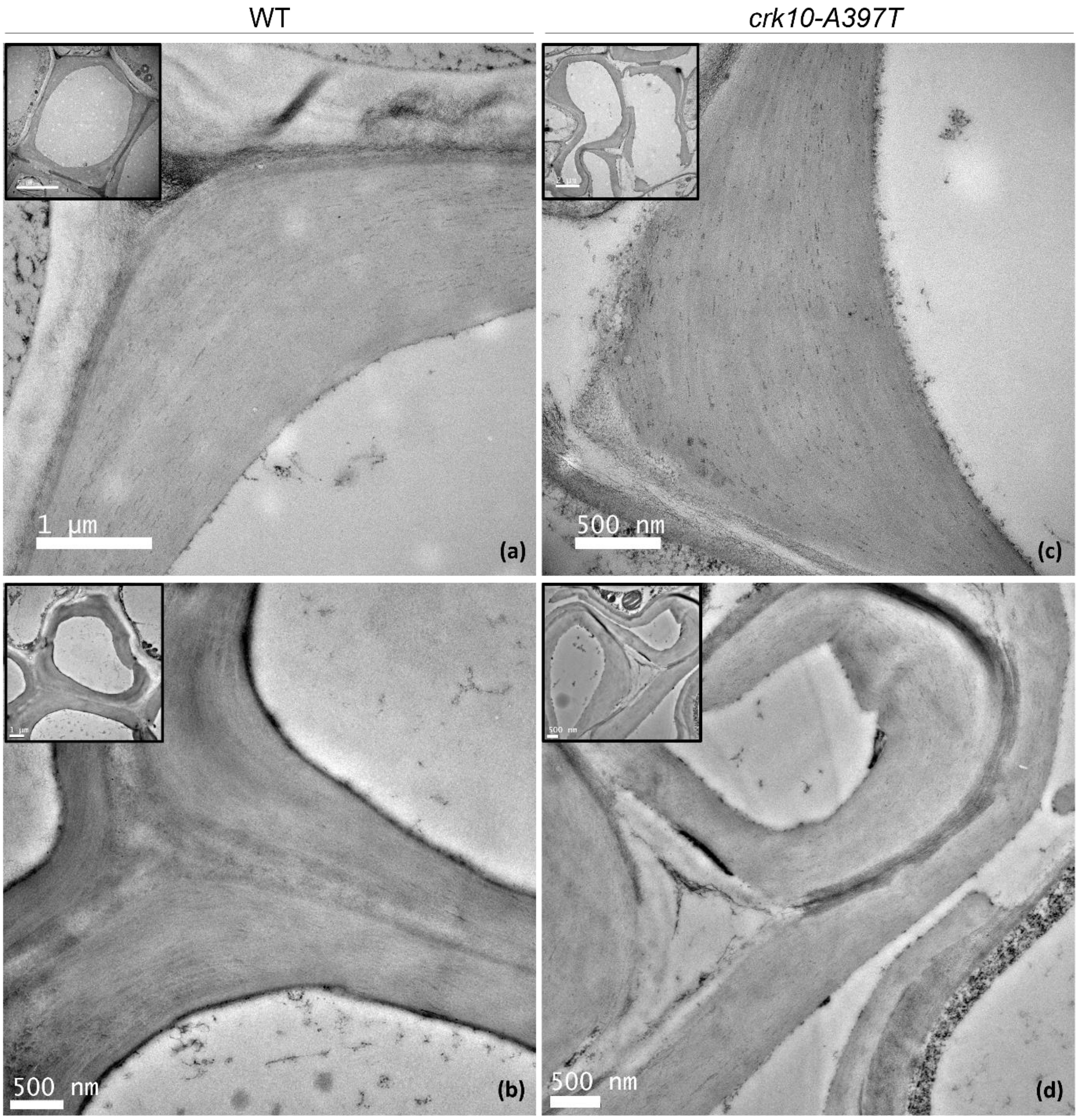

Supplementary Figure S13 The ultrastructure of the secondary cell wall of collapsed xylem vessels resembles that of intact vessels in the WT.

(A-D) TEM micrographs of cross sections of hypocotyls of 3-week-old WT (A, B) and *crk10-A397T* (C, D) plants showing secondary cell wall of xylem. Inserts at top left corner show lower magnification of the imaged area. Three biological replicates were analysed per genotype. Bars, (A, insert top left corner B) = 1 µm; (B, C, D, insert top left corner D) = 500 nm; insert top left corner A = 5 µm; insert top left corner C = 2 µm.

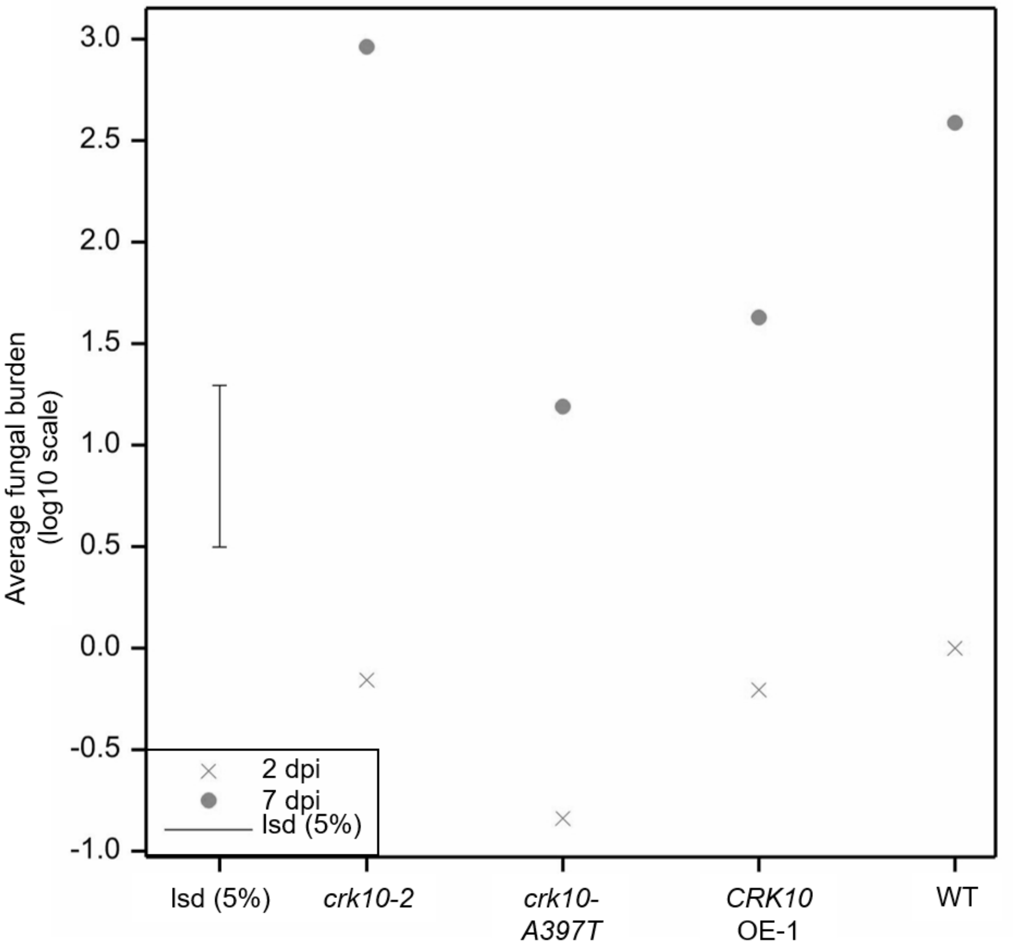

Supplementary Figure S14 Fungal burden quantification at 2 and 7 days post inoculation with *F. oxysporum*. Fungal burden determined by the relative amount of fungal (*F. oxysporum ACTIN1* gene) to plant (Arabidopsis *ACTIN2* gene) DNA. Graph shows means of three independent experiments; least significant difference (l.s.d) bar is shown.

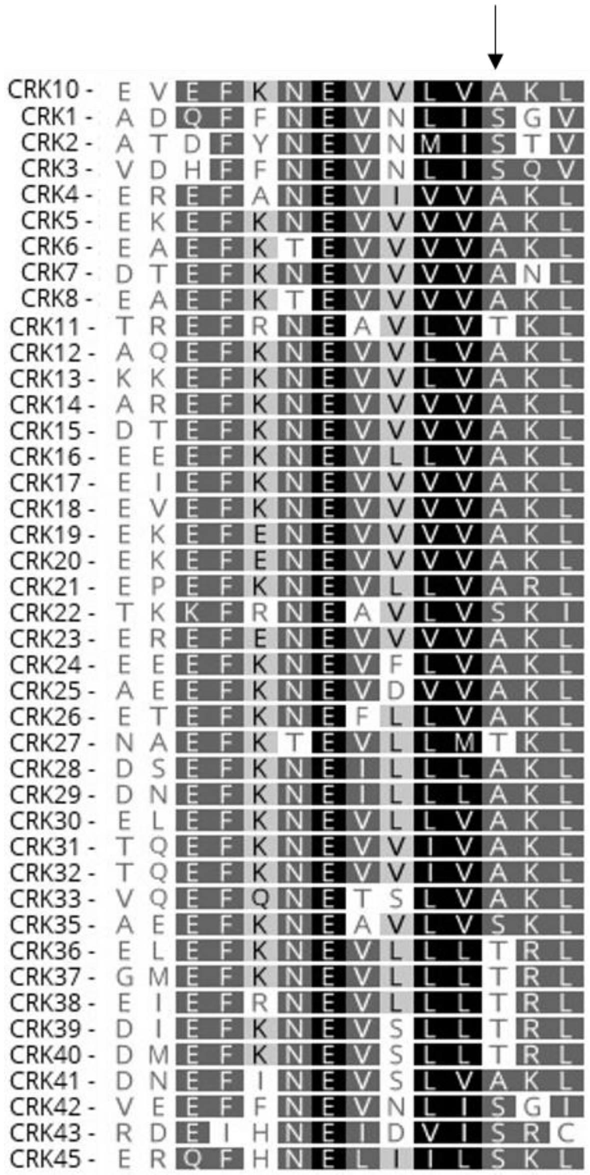

Supplementary Figure S15 Alignment of the αC-helix segment of the kinase domain of the CRK family from *A. thaliana* shows members of the family which contain alanine, threonine or serine residues on position equivalent to Ala397 in *CRK10*. Amino acid sequences of the subdomain III / αC-helix of the kinase domain of 42 members of the CRK family from *A. thaliana* were imported from Uniprot (Uniprot Consortium; uniprot.org) and aligned used the Geneious Alignment tool (Geneious 10.0.9); alignment type: global alignment with free end gaps; cost matrix: Blosum62. Black arrow indicates Ala397 position in CRK10.

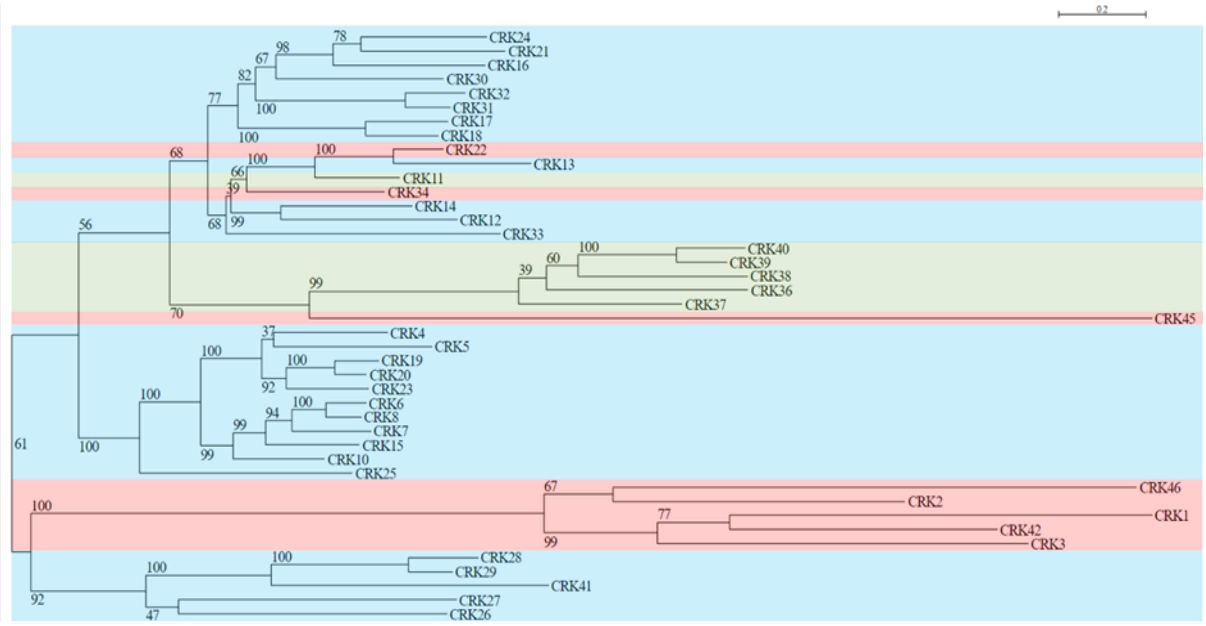

Supplementary Figure S16 Phylogenetic tree of the *CRK* family from *A. thaliana* and their respective residue on position equivalent to Ala397 in *CRK10*. Amino acid sequences of 42 members of the CRK family from *A. thaliana* were imported from Uniprot (Uniprot Consortium; uniprot.org). The 42 sequences were aligned using the MAFFT online tool (Katoh et al., 2019; v7.505) with the following parameters: scoring matrix Blosum62, gap opening penalty 1.53 with iteration. The maximum likelihood tree was built using PhyML (Guindon et al., 2010; v3.3.20200621), substitution model LG; the reliability of internal branches was assessed using bootstrap 100x. CRKs highlighted in red contain a serine at the position equivalent to Ala397 of *CRK10*, CRKs with a threonine are highlighted in green and CRKs with an alanine in blue.

Supplementary Table S1. Table of primers.

**Primers used to generate the 35S:*CRK10*-NOS construct (5'-3')**		**Purpose**
CRK10 *Sal*I For	GTCGACATGAGAAGAAACACAGATCA	Amplification of *CRK10* cDNA from clone U60398

CRK10 *Sac*I Rev	GAGCTCTCATCGAGGATGTATATCTGTG	Amplification of *CRK10* cDNA from clone U60398
35S *Asc*I For	GGCGCGCCATGCCTGCAGGCTCT	Amplification of 35S:*CRK10*:NOS for cloning into binary vector RS 3GSeedDSRed MCS
NOSt *Asc*I Rev	GGCGCGCCTATGACATGATTA	Amplification of S35:*CRK10*:NOS for cloning into binary vector RS 3GSeedDSRed MCS
**Primers used to generate *CRK10*$_{\mathbf{Pro}}$:*crk10-A397T*-NOS**		**Purpose**
CRK10 Pro *Sph*I For	GCATGCCCTTTGCAACTAGCTAGATGGA	Amplification of 1kb *CKR10* promoter from genomic DNA
CRK10 Pro *Sal*I Rev	GTCGACAGCTTTGAGTGATATATGAA	Amplification of 1kb *CKR10* promoter from genomic DNA
CRK10 A397T For	GAGGTTGTTCTTGTTACAAAGCTACAACATAGA	*In vitro* mutagenesis (introduce A397T mutation in CRK10)
CRK10 A397T Rev	TCTATGTTGTAGCTTTGTAACAAGAACAACCTC	*In vitro* mutagenesis (introduce A397T mutation in CRK10)
CRK10 Pro *Asc*I For	GGCGCGCCTTTGCAACTAGCTAGATGGA	Amplification of CRK10$_{Pro}$:*crk10-A397T*-NOS for cloning into binary vector RS 3GSeedDRed MCS
NOSt *Asc*I Rev	GGCGCGCCTATGACATGATTA	Amplification of CRK10$_{Pro}$:*crk10-A397T*-NOS for cloning into binary vector RS 3GSeedDSRed MCS
**Primers used to generate reporter constructs (CRK10$_{\mathbf{Pro}}$:*GUS*-NOS and 35S:*CRK10-mCherry*-NOS)**		**Purpose**
CRK10 Pro *Sph*I For	GCATGCCCTTTGCAACTAGCTAGATGGA	Amplification of 1kb *CKR10* promoter from genomic DNA
CRK10 Pro *Nco*I Rev	CCATGGAGCTTTGAGTGATATATGAA	Amplification of 1kb *CKR10* promoter from genomic DNA
CRK10 Pro *Asc*I For	GGCGCGCCTTTGCAACTAGCTAGATGGA	Amplification of CRK10$_{Pro}$:*GUS*-NOS for cloning into binary vector RS 3GSeedDSRed MCS
NOSt *Asc*I Rev	GGCGCGCCTATGACATGATTA	Amplification of CRK10$_{Pro}$:*GUS*-NOS for cloning into binary vector RS 3GSeedDSRed MCS
CRK10 wsc *Sac*I For	GATATACATCCTCGAGAGCTCCTCGAATTGATCG TT	*In vitro* mutagenesis (replace CRK10 stop codon with *Sac*I site in pJD330 35S:*CRK10*-tNOS)

CRK10 wsc *Sac*I Rev	AACGATCAATTCGAGGAGCTCTCGAGGATGTATATC	*In vitro* mutagenesis (replace CRK10 stop codon with *Sac*I site in pJD330 35S:*CRK10*-tNOS)
mCherry *Sac*I For	GAGCTCATGGTGAGCAAGG	Amplification of *mCherry*
mCherry *Sac*I Rev	GAGCTCTCATTGCCAAATGTTTG	Amplification of *mCherry*
CRK10 *Sal*I For	GTCGACATGAGAAGAAACACAG	Amplification of *CRK10-mCherry*-NOS for cloning into pENTR1A.
NOSt *Not*I Rev	GGCGCGCCTATGACATGATTACGAATTC	Amplification of *CRK10-mCherry*-NOS for cloning into pENTR1A.
**Primers used to generate His-tagged kinase domain constructs**		**Purpose**
CRK10 KD *Sal*I For	GTCGACATGGATGATATCACAAC	Amplification of the kinase domain of CRK10
CRK10 KD *Not*I Rev	GCGGCCGCTTATCGAGGAT	Amplification of the kinase domain of CRK10
CRK10 A397T For	GAGGTTGTTCTTGTTACAAAGCTACAACATAGA	*In vitro* mutagenesis (introduce A397T mutation in the kinase domain of CRK10)
CRK10 A397T Rev	TCTATGTTGTAGCTTTGTAACAAGAACAACCTC	*In vitro* mutagenesis (introduce A397T mutation in the kinase domain of CRK10)
CRK10 D473N For	ACAATCATACACCGTAATCTCAAAGCCAGTAAC	*In vitro* mutagenesis (generation of dead kinase version of the CRK10 kinase domain)
CRK10 D473N Rev	GTTACTGGCTTTGAGATTACGGTGTATGATTGT	*In vitro* mutagenesis (generation of dead kinase version of the CRK10 kinase domain)
**Primers used for quantification of transcript abundance by quantitative PCR**		**Purpose**
AtCRK10 For	ACATGTCTCCCGAGTATGCAATG	qPCR
AtCRK10 Rev	CCAAAGCCCCCAAGCATATGAG	qPCR
AtACT2 For	TTCCCTCAGCACATTCCAGCAGAT	qPCR
AtACT2 Rev	AACGATTCCTGGACCTGCCTCATC	qPCR
AtUBC21 For	GCTCTTATCAAAGGACCTTCGG	qPCR
AtUBC21 Rev	CGAACTTGAGGAGGTTGCAAAG	qPCR
**Primers used for fungal burden quantification by quantitative PCR**		**Purpose**
ACTIN1 For (*F. oxysporum*)	ATGTCACCACCTTCAACTCCA	qPCR

ACTIN1 Rev (*F. oxysporum*)	CTCTCGTCGTACTCCTGCTT	qPCR
AtACT2 For	TTCCCTCAGCACATTCCAGCAGAT	qPCR
AtACT2 Rev	AACGATTCCTGGACCTGCCTCATC	qPCR

SupplementaryTables S2–S5. Differentially expressed genes in the crk10-A397T mutant hypocotyls.

Supplementary Table S2. Differentially expressed genes (DEGs) in the hypocotyl of the *crk10-A397T* plants (two weeks after sowing).

Supplementary Table S3. Differentially expressed genes (DEGs) in the hypocotyl of the *crk10-A397T* plants (three weeks after sowing).

Supplementary Table S4. Differentially expressed genes (DEGs) in the hypocotyl of the *crk10-A397T* plants (five weeks after sowing).

Supplementary Table S5. Core differentially expressed genes in the *crk10-A397T* plants (all time points).

Supplementary Tables S6, S7. Gene Ontology analysis.

Supplementary Table S6. AgriGO results - Single Enrichment Tool for UP-REGULATED CORE DEGs - *crk10-A397T* mutant.

Supplementary Table S7. AgriGO results - Single Enrichment Tool for DOWN-REGULATED CORE DEGs - *crk10-A397T* mutant.

Tables S8–S11. Tables of differentially expressed genes per functional category.

Supplementary Table S8. Transcriptional induction of genes involved defence responses to pathogens and salicylic acid signalling in the hypocotyls of *crk10-A397T* mutant plants.

		**Log2 Fold Change**		
**Gene ID**	**Gene name**	**2 weeks**	**3 weeks**	**5 weeks**
AT2G14610	*PATHOGENESIS-RELATED 1 (PR1)*	-	3.98	4.46
AT3G57260	*PATHOGENESIS-RELATED 2 (PR2)*	4.25	6.84	5.99
AT3G12500	*PATHOGENESIS-RELATED 3 (PR3)*	2.04	3.68	3.23
AT3G04720	*PATHOGENESIS-RELATED 4 (PR4)*	-	2.17	-
AT1G75040	*PATHOGENESIS-RELATED 5 (PR5)*	5.53	7.71	5.30
AT2G30750	*CYP71A12*	4.48	4.50	5.80
AT2G30770	*CYP71A13*	9.20	7.01	8.74
AT3G26830	*PHYTOALEXIN DEFICIENT 3 (PAD3)*	6.50	6.45	4.51

AT3G52430	*PHYTOALEXIN DEFICIENT 4 (PAD4)*	2.29	-	1.77
AT1G19250	*FLAVIN-DEPENDENT MONOOXYGENASE 1 (FMO1)*	2.63	4.75	7.48
AT3G48090	*ENHANCED DISEASE SUSCEPTIBILITY 1 (EDS1)*	1.88	1.79	2.04
AT5G54610	*ANKYRIN / BDA1*	2.68	3.43	6.8
AT5G13320	*AVRPPHB SUSCEPTIBLE 3 (PBS3)*	4.13	4.52	6.22
AT5G40990	*GDSL LIPASE 1 (GLIP1)*	6.20	6.30	2.59
AT2G31880	*SUPPRESSOR OF BIR 1 (SOBIR1)*	2.20	2.59	2.34
AT1G32960	*SUBTILASE 3.3 (SBT3.3)*	4.01	7.88	3.43
AT1G21120	*INDOLE GLUCOSINOLATE O-METHYLTRANSFERASE 2*	3.32	3.83	2.98
AT1G21110	*INDOLE GLUCOSINOLATE O-METHYLTRANSFERASE 3*	3.32	4.49	3.19
AT5G52810	*SAR DEFICIENT 4 (SARD4)*	1.96	-	2.94
AT4G16890	*SUPPRESSOR OF NPR1-1, CONSTITUTIVE 1 (SNC1)*	-	1.61	-

Supplementary Table S9. Differentially expressed transcription factors in the *crk10-A397T* mutant transcriptome.

**UP-REGULATED GENES**				
		**Log2 Fold Change**		
**Gene ID**	**Gene name**	**2 weeks**	**3 weeks**	**5 weeks**
AT1G01010	*NAC001*	2.45	2.59	2.05
AT1G01720	*NAC002 / ATAF1*	-	2.15	-
AT3G29035	*NAC003 / NAC059*	-	3.20	-
AT1G32870	*NAC013*	-	2.35	-
AT1G34180	*NAC016*	1.99	3.84	-
AT1G77450	*NAC032*	-	2.59	-
AT2G17040	*NAC036*	1.97	3.26	1.91
AT2G43000	*NAC042*	3.53	4.60	-
AT3G04420	*NAC048*	-	2.32	-
AT3G10500	*NAC053*	-	1.86	-

AT3G44350	*NAC061*	-	4.71	-
AT4G27410	*NAC072*	-	2.34	-
AT5G08790	*NAC081 / ATAF2*	-	2.65	2.02
AT5G14490	*NAC085*	-	5.74	-
AT5G22380	*NAC090*	3.88	3.97	3.15
AT5G63790	*NAC102*	-	2.65	-
AT1G62300	*WRKY6*	2.05	3.08	3.66
AT2G23320	*WRKY15*	-	1.53	-
AT5G07100	*WRKY26*	-	2.80	-
AT5G24110	*WRKY30*	4.99	5.74	2.78
AT4G22070	*WRKY31*	-	3.78	-
AT5G22570	*WRKY38*	-	-	4.21
AT4G11070	*WRKY41*	4.90	5.34	4.75
AT3G01970	*WRKY45*	2.37	3.27	-
AT5G26170	*WRKY50*	4.53	3.77	3.29
AT5G64810	*WRKY51*	3.16	3.82	4.68
AT4G23810	*WRKY53*	1.91	2.73	-
AT2G40740	*WRKY55*	3.55	4.05	-
AT2G25000	*WRKY60*	-	3.36	1.79
AT5G01900	*WRKY62*	-	-	4.38
AT3G56400	*WRKY70*	-	-	2.04
AT5G13080	*WRKY75*	2.64	4.72	3.77
AT2G47190	*MYB2*	2.79	-	-
AT2G31180	*MYB14*	-	-	3.72
AT3G28910	*MYB30*	-	3.50	3.41
AT1G18710	*MYB47*	-	8.54	-
AT1G18570	*MYB51*	-	3.07	-
AT5G59780	*MYB59*	-	1.89	-
AT1G09540	*MYB61*	-	2.09	-
AT1G68320	*MYB62*	-	5.76	-
AT2G02820	*MYB88*	-	2.63	-
AT3G47600	*MYB94*	-	2.90	-
AT1G48000	*MYB112*	-	5.83	-
AT3G30210	*MYB121*	-	6.32	-
AT1G74080	*MYB122*	4.90	4.47	-

AT3G23150	*ETHYLENE RESPONSE FACTOR 2 (ERF2)*	1.84	-	-
AT1G04370	*ETHYLENE-RESPONSIVE ELEMENT BINDING FACTOR 14 (ERF14)*	5.67	4.11	6.45
AT2G47520	*ETHYLENE RESPONSE FACTOR 71 (ERF71)*	3.95	2.51	2.95
AT5G61890	*ETHYLENE RESPONSE FACTOR 114 (ERF114)*	-	-	3.27
AT5G07310	*ETHYLENE RESPONSE FACTOR 115 (ERF115)*	-	4.97	-
AT5G13330	*RELATED TO AP2 6L / ETHYLENE RESPONSE FACTOR*	-	3.63	3.07
**DOWN-REGULATED GENES**				
		**Log2 Fold Change**		
**Gene ID**	**Gene name**	**2 weeks**	**3 weeks**	**5 weeks**
AT3G61910	*NAC066*	-	-	-2.26
AT4G28530	*NAC074*	-	-3.21	-
AT5G46590	*NAC096*	-	-2.60	-
AT1G30650	*WRKY14*	-	-	-2.21
AT2G21650	*RAD-LIKE 2 / MYB1*	-3.72	-6.85	-6.07
AT4G36570	*RAD-LIKE 3*	-4.15	-9.19	-5.34
AT2G18328	*RAD-LIKE 4*	-4.36	-9.33	-5.37
AT1G75250	*RAD-LIKE 6 / MYB3*	-3.11	-7.68	-3.79
AT2G37260	*WRKY44*	-	-1.99	-
AT3G12820	*MYB10*	-	-1.85	-
AT4G25560	*MYB18*	-	-3.98	-
AT3G13890	*MYB26*	-	-	-5.70
AT5G61420	*MYB28*	-	-2.67	-2.87
AT5G60890	*MYB34*	-	-2.24	-
AT5G16600	*MYB43*	-	-	-2.03
AT1G16490	*MYB58*	-	-	-2.19
AT5G49330	*MYB111*	-5.22	-	-
AT5G35550	*MYB123*	-	-	-2.95
AT1G14350	*MYB124*	-	-	-1.57

AT2G44940	*ETHYLENE RESPONSE FACTOR 34 (ERF34)*	-	-1.64	-
AT3G60490	*ETHYLENE RESPONSE FACTOR 35 (ERF35)*	-	-1.64	-
AT5G25810	*ETHYLENE RESPONSE FACTOR 40*	-	-2.24	-
AT3G23230	*ETHYLENE RESPONSE FACTOR 98*	-2.29	-4.90	-
AT4G34410	*ETHYLENE RESPONSE FACTOR 109 (ERF109)*	-3.55	-	-
AT4G36920	*APETALA2*	-	-1.82	-

Supplementary Table S10. Differentially expressed genes in the *crk10-A397T* mutant transcriptome associated with abscisic acid (ABA).

**UP-REGULATED GENES**				
		**Log2 Fold Change**		
**Gene ID**	**Gene name**	**2 weeks**	**3 weeks**	**5 weeks**
AT1G30100	*9-CIS-EPOXYCAROTENOID DIOXYGENASE (NCED5)*	4.26	7.26	-
AT1G66600	*ABA OVERLY SENSITIVE 3 (ABO3) / WRKY63*	3.73	2.30	6.26
AT1G65690	*NDR1/HIN1-LIKE PROTEIN 6 (NHL6)*	1.69	2.82	2.35
AT4G11890	*ABA- AND OSMOTIC-STRESS-INDUCIBLE RECEPTOR-LIKE CYTOSOLIC KINASE1(ARCK1) / CRK45*	2.07	3.72	3.27
AT1G15520	*ATP-BINDING CASSETTE G40 (ABCG40)*	5.68	9.64	7.75
AT1G69850	*ABA-IMPORTING TRANSPORTER 1 (AIT1)*	-	1.99	-
AT5G57050	*ABSCISIC ACID-INSENSITIVE 2 (ABI2)*	-	1.94	-
AT2G36270	*ABSCISIC ACID-INSENSITIVE 5 (ABI5)*	-	2.34	-
AT4G34220	*RECEPTOR DEAD KINASE 1*	-	1.56	-
AT5G04760	*DIVARICATA2*	-	2.25	-
AT2G26300	*G PROTEIN ALPHA SUBUNIT 1 (GP ALPHA 1)*	-	1.43	-

AT3G27250	*ABA-INDUCED TRANSCRIPTION REPRESSOR 1 (AITR1)*	5.10	5.42	-
AT5G40800	*ABA-INDUCED TRANSCRIPTION REPRESSOR 4 (AITR4)*	-	7.32	-
AT1G18100	*MOTHER OF FT AND TFL1 (MFT)*	-	5.08	-
AT5G15960	*KIN1*	2.73	-	-
AT5G59220	*HIGHLY ABA-INDUCED PP2C GENE 1 (HAI1) / SENESCENCE ASSOCIATED GENE 113 (SAG113)*	-	7.86	-
AT4G21680	*NITRATE TRANSPORTER 1.8 (NRT1.8)*	-	2.98	-
AT5G66400	*RESPONSIVE TO ABA 18 (RAB18)*	-	5.49	-
AT3G21780	*UDP-GLUCOSYL TRANSFERASE 71B6 (UGT71B6)*	-	2.99	-
**DOWN-REGULATED GENES**				
		**Log2 Fold Change**		
**Gene ID**	**Gene name**	**2 weeks**	**3 weeks**	**5 weeks**
AT1G01360	*REGULATORY COMPONENT OF ABA RECEPTOR 1 (RCAR1)*	-	-1.64	-2.19
AT5G53160	*REGULATORY COMPONENT OF ABA RECEPTOR 3 (RCAR3)*	-	-1.81	-

Supplementary Table S11. Differentially expressed genes in the *crk10-A397T* mutant transcriptome associated cell wall biosynthesis / modification and xylem vessel formation.

**UP-REGULATED GENES**				
		**Log2 Fold Change**		
**Gene ID**	**Gene name**	**2 weeks**	**3 weeks**	**5 weeks**
AT5G05340	*PEROXIDASE 52 (PRX52)*	4.99	5.48	-
AT5G51890	*PEROXIDASE 66 (PRX66)*	3.91	6.52	6.79
AT4G08770	*PEROXIDASE 37 (PER37)*	3.58	-	2.79
AT5G60020	*LACCASE 17 (LAC17)*	3.16	3.97	-
AT2G29130	*LACCASE 2 (LAC2)*	4.28	2.15	-
AT5G03260	*LACCASE 11 (LAC11)*	-	2.50	2.87
AT3G50220	*IRREGULAR XYLEM 15 (IRX15)*	2.11	2.33	-

AT3G18660	*GLUCURONIC ACID SUBSTITUTION OF XYLAN 1 (GUX1)*	1.89	2.64	-
AT1G33800	*GLUCURONOXYLAN METHYLTRANSFERASE 1 (GXMT1)*	-	2.3	-
AT5G54690	*IRREGULAR XYLEM 8 (IRX8)*	-	1.59	-
AT5G44030	*CELLULOSE SYNTHASE A4 (CESA4) / IRREGULAR XYLEM 5 (IRX5)*	-	1.98	-
AT2G28110	*FRAGILE FIBER 8 (FRA8) / IRREGULAR XYLEM 7 (IRX7)*	-	1.66	-
AT3G56000	*CELLULOSE SYNTHASE LIKE A14*	-	2.77	3.65
AT1G55850	*CELLULOSE SYNTHASE LIKE E1*	-	2.64	-
AT4G24000	*CELLULOSE SYNTHASE LIKE G2*	-	6.70	-
AT1G69530	*EXPANSIN 1*	-	1.81	2.88
AT1G26770	*EXPANSIN 10*	-	4.49	-
AT1G21310	*EXTENSIN 3*	-	2.29	3.26
AT3G47380	*PECTIN METHYLESTERASE INHIBITOR 11 (PMEI11)*	4.24	4.39	5.34
AT1G57590	*PECTIN ACETYLESTERASE 2*	-	5.24	-
AT3G59010	*PECTIN METHYLESTERASE (PME35)*	-	2.34	-
AT4G12390	*PECTIN METHYLESTERASE INHIBITOR 1*	-	1.86	-
AT1G70500	Pectin lyase-like superfamily protein	1.97	2.81	2.76
AT2G43870	Pectin lyase-like superfamily protein	4.04	6.25	4.1
AT3G24130	Pectin lyase-like superfamily protein	-	4.38	-
AT3G28180	*XYLOGLUCAN GLYCOSYLTRANSFERASE 4*	-	2.95	-
AT4G25810	*XYLOGLUCAN ENDOTRANSGLUCOSYLASE 6 (XTR6)*	-	3.27	-
AT3G48580	*XYLOGLUCAN HYDROLASE 11 (XTH11)*	-	4.61	3.46
AT4G18990	*XYLOGLUCAN HYDROLASE 29 (XTH29)*	-	5.49	-
AT3G29810	*COBRA-LIKE PROTEIN 2 PRECURSOR (COBL2)*	-	3.01	
AT4G35350	*XYLEM CYSTEINE PROTEASE 1 (XCP1)*	-	1.50	2.59
AT1G20850	*XYLEM CYSTEINE PROTEASE 2 (XCP2)*	-	-	1.67

AT2G31110	*TRICHOME BIREFRINGENCE-LIKE 40*	1.68	2.42	-
AT4G08160	*ENDO-1,4-BETA-XYLANASE 3*	-	2.12	-
AT1G10050	*ENDO-1,4-BETA-XYLANASE 2*	-	1.57	-
AT5G66690	*UGT72E2*	-	4.02	2.28
AT3G24503	*ALDEHYDE DEHYDROGENASE 1A*	-	2.47	2.75
AT1G71380	*ARABIDOPSIS THALIANA GLYCOSYL HYDROLASE 9B3*	-	3.05	-
AT5G06230	*TRICHOME BIREFRINGENCE-LIKE 9*	-	2.06	-
**DOWN-REGULATED GENES**				
		**Log2 Fold Change**		
**Gene ID**	**Gene name**	**2 weeks**	**3 weeks**	**5 weeks**
AT2G22900	*MUCILAGE-RELATED 10*	-	-1.42	-
AT5G65810	*COTTON GOLGI RELATED 3*	-	-1.50	-
AT3G14310	*PECTIN METHYLESTERASE 3 (PME3)*	-	-2.58	-
AT5G62360	*PECTIN METHYLESTERASE INHIBITOR 13 (PMEI13)*	-	-3.55	-
AT4G23820	Pectin lyase-like superfamily protein	-	-2.68	-
AT3G62110	Pectin lyase-like superfamily protein	-	-1.56	-
AT5G48900	Pectin lyase-like superfamily protein	-1.62	-3.65	-
AT5G19730	Pectin lyase-like superfamily protein	-	-1.57	-
AT1G04680	Pectin lyase-like superfamily protein	-	-1.32	-
AT3G61490	Pectin lyase-like superfamily protein	-	-2.08	-
AT5G04310	Pectin lyase-like superfamily protein	-	-2.39	-
AT4G23500	Pectin lyase-like superfamily protein	-	-1.57	-
AT5G09760	Plant invertase/pectin methylesterase inhibitor superfamily	-	-1.41	-
AT2G26450	Plant invertase/pectin methylesterase inhibitor superfamily	-	-3.73	-
AT1G56100	Plant invertase/pectin methylesterase inhibitor superfamily protein	-	-6.23	-
AT5G20860	Plant invertase/pectin methylesterase inhibitor superfamily	-	-2.14	-
AT1G23205	Plant invertase/pectin methylesterase inhibitor superfamily protein	-	-2.95	-
AT3G62820	Plant invertase/pectin methylesterase inhibitor superfamily protein	-	-1.92	-

AT4G16980	Arabinogalactan-protein family	-	-3.54	-
AT1G71690	*GLUCURONOXYLAN 4-O-METHYLTRANSFERASE-LIKE PROTEIN (DUF579)*	-	-2.05	-
AT1G67830	*ALPHA-FUCOSIDASE 1*	-	-1.69	-
AT5G08370	*ALPHA-GALACTOSIDASE 2*	-	-1.74	-
AT5G26120	*ALPHA-L-ARABINOFURANOSIDASE 2*	-	-4.06	-
AT5G40730	*ARABINOGALACTAN PROTEIN 24*	-	-2.22	-
AT1G28290	*ARABINOGALACTAN PROTEIN 31*	-	-2.08	-
AT5G24105	*ARABINOGALACTAN PROTEIN 41*	-	-3.66	-
AT5G64570	*BETA-D-XYLOSIDASE 4*	-	-3.19	-
AT5G63810	*BETA-GALACTOSIDASE 10*	-	-2.34	-
AT3G52840	*BETA-GALACTOSIDASE 2*	-	-2.20	-
AT4G21760	*BETA-GLUCOSIDASE 47*	-1.88	-5.04	-2.07
AT1G02640	*BETA-XYLOSIDASE 2*	-	-2.53	-
AT2G13680	*CALLOSE SYNTHASE 5*	-	-2.80	-
AT1G22880	*CELLULASE 5*	-	-2.40	-
AT1G77460	*CELLULOSE SYNTHASE INTERACTIVE 3*	-	-1.51	-
AT1G24070	*CELLULOSE SYNTHASE-LIKE A10*	-	-2.58	-2.22
AT4G07960	*CELLULOSE SYNTHASE-LIKE C12*	-	-2.71	-
AT4G01630	*EXPANSIN A17*	-	-1.94	-
AT3G29030	*EXPANSIN A5*	-	-5.23	-
AT2G28950	*EXPANSIN A6*	-	-2.00	-
AT2G40610	*EXPANSIN A8*	-	-3.94	-
AT4G28250	*EXPANSIN B3*	-	-3.34	-
AT5G55730	*FASCICLIN-LIKE ARABINOGALACTAN 1*	-	-1.58	-
AT2G45470	*FASCICLIN-LIKE ARABINOGALACTAN PROTEIN 8*	-	-1.36	-
AT1G71990	*FUCOSYLTRANSFERASE 13*	-	-1.98	-
AT1G74420	*FUCOSYLTRANSFERASE 3*	-	-2.20	-
AT1G13250	*GALACTURONOSYLTRANSFERASE-LIKE 3*	-	-1.85	-
AT5G58910	*LACCASE 16*	-	-2.21	-

AT2G30210	*LACCASE 3*	-	-1.88	-
AT2G22900	*MANNAN α151 GALACTOSYL TRANSFERASE 1*	-	-1.42	-
AT5G15900	*TRICHOME BIREFRINGENCE-LIKE 19*	-	-2.72	-
AT1G60790	*TRICHOME BIREFRINGENCE-LIKE 2*	-	-1.59	-
AT2G40150	*TRICHOME BIREFRINGENCE-LIKE 28*	-	-1.80	-
AT2G30010	*TRICHOME BIREFRINGENCE-LIKE 45*	-	-2.57	-
AT3G62390	*TRICHOME BIREFRINGENCE-LIKE 6*	-	-1.74	-
AT2G36870	*XYLOGLUCAN ENDOTRANSGLUCOSYLASE / HYDROLASE 32*	-	-2.17	-
AT4G37800	*XYLOGLUCAN ENDOTRANSGLUCOSYLASE / HYDROLASE 7*	-	-1.92	-

Supplementary Table S12. Quantification of hormones in the hypocotyl of WT and *crk10-A397T* mutant plants. Average values for three biological replicates (± standard error). Asterisks indicate statistical significance (t-test): $^{**} = p \leq 0.01$; $^{***} = p \leq 0.001$.

	Quantification (ng/g hypocotyl)		
	**ABA**	**SA**	**JA**
WT	8.523 ±0.435	337.120 ±13.760	7.673 ±2.103
crk10-A397T	13.293** ±0.434	977.837*** ±65.434	4.743 ±1.713
